# Insulin resistance disrupts epithelial repair and niche-progenitor Fgf signaling during chronic liver injury

**DOI:** 10.1371/journal.pbio.2006972

**Published:** 2019-01-29

**Authors:** Fátima Manzano-Núñez, María José Arámbul-Anthony, Amparo Galán Albiñana, Aranzazu Leal Tassias, Carlos Acosta Umanzor, Irene Borreda Gascó, Antonio Herrera, Jerónimo Forteza Vila, Deborah J. Burks, Luke A. Noon

**Affiliations:** 1 CIBERDEM (Centro de Investigación Biomédica en Red de Diabetes y Enfermedades Metabólicas Asociadas), Madrid, Spain; 2 Centro de Investigación Príncipe Felipe, Valencia, Spain; 3 Instituto Valenciano de Patología, Universidad Católica de Valencia San Vicente Màrtir, Valencia, Spain; University of Pennsylvania, United States of America

## Abstract

Insulin provides important information to tissues about feeding behavior and energy status. Defective insulin signaling is associated with ageing, tissue dysfunction, and impaired wound healing. In the liver, insulin resistance leads to chronic damage and fibrosis, but it is unclear how tissue-repair mechanisms integrate insulin signals to coordinate an appropriate injury response or how they are affected by insulin resistance. In this study, we demonstrate that insulin resistance impairs local cellular crosstalk between the fibrotic stroma and bipotent adult liver progenitor cells (LPCs), whose paracrine interactions promote epithelial repair and tissue remodeling. Using insulin-resistant mice deficient for insulin receptor substrate 2 (*Irs2*), we highlight dramatic impairment of proregenerative fibroblast growth factor 7 (Fgf7) signaling between stromal niche cells and LPCs during chronic injury. We provide a detailed account of the role played by IRS2 in promoting Fgf7 ligand and receptor (Fgfr2-IIIb) expression by the two cell compartments, and we describe an insulin/IRS2-dependent feed-forward loop capable of sustaining hepatic re-epithelialization by driving FGFR2-IIIb expression. Finally, we shed light on the regulation of IRS2 and FGF7 within the fibrotic stroma and show—using a human coculture system—that *IRS2* silencing shifts the equilibrium away from paracrine epithelial repair in favor of fibrogenesis. Hence, we offer a compelling insight into the contribution of insulin resistance to the pathogenesis of chronic liver disease and propose IRS2 as a positive regulator of communication between cell types and the transition between phases of stromal to epithelial repair.

## Author summary

“Insulin resistance” is a chronic state of reduced sensitivity to the effects of circulating insulin. It is one of the hallmarks of metabolic disease and a consequence of ageing, but insulin resistance is also observed in otherwise healthy individuals after severe trauma/hemorrhage/sepsis, suggesting that it plays a physiological role in modulating the response to injury. Defective insulin signals are linked to impaired wound healing, yet it remains unclear how systemic changes affect locally the cells that coordinate tissue repair. In this study, we used the liver to assess how insulin resistance impacts the injury response in mice. We provide proof of concept that insulin signals are locally integrated by the fibrotic microenvironment surrounding the adult liver stem cells during chronic injury, resulting in the increased expression of epithelial repair signals. Insulin also simultaneously primes stem cells to respond to these stromal growth factors, leading to an increased participation in epithelial repair. Insulin resistance disrupts this local paracrine circuit, resulting in a blunted epithelial response to chronic injury that exacerbates tissue damage. Our model highlights a potential role for insulin in switching the hepatic injury response from a stromal repair process to an epithelial repair process. To our knowledge, our data provide a new perspective from which to reassess how insulin resistance influences fibrosis, wound healing, and tissue remodeling during injury.

## Introduction

Successful wound healing requires coordination between stromal and epithelial cell compartments. Stromal activation lays the groundwork for epithelial repair, producing the appropriate microenvironment and growth factors to facilitate proliferation and remodeling of epithelia [1]. Metabolic disease is associated with a spectrum of chronic comorbidities, including cardiovascular disease and liver disease, as well as defects in wound healing [2,3]. However, it remains unclear whether insulin resistance affects injury-repair mechanisms in target organs such as the liver, which are otherwise at the forefront of insulin's metabolic actions. The liver parenchyma is highly regenerative and can undergo dramatic tissue remodeling to maintain parenchymal function in the face of chronic injury. Such remodeling is shaped in part by the activation of perisinusoidal cells, such as hepatic stellate cells (HSCs), and periportal mesenchymal cells, such as portal fibroblasts (PFs) [4], that expand to produce a fibrotic milieu capable of directing epithelial repair but that also contribute to long-term risk of scarring/fibrosis and hepatic dysfunction. To date, the impact of insulin resistance on the fibrotic stroma, including how it affects the ability of mesenchymal cells to communicate repair signals to the hepatic epithelia, remains unknown [5].

In this study, we investigate how insulin resistance affects stromal–epithelial repair mechanisms in the liver during chronic injury by knockout of insulin receptor substrate 2 (*Irs2*), a key adaptor protein that couples the insulin and insulin-like growth factor 1 (IGF-1) receptors to intracellular signaling pathways. *Irs2*^−/−^ mice have normal liver development but severe peripheral insulin resistance that leads to late-onset type II diabetes [6]. IRS2 is the principle regulator of insulin sensitivity in hepatocytes [7], cooperating closely with IRS1 to mediate the metabolic response to feeding [8]. Aberrant IRS2 expression has been associated with hepatic insulin resistance [9] and progression of chronic liver diseases, including nonalcoholic steatohepatitis (NASH) [10], hepatitis C [11], and hepatocellular carcinoma [12], in which increased IRS2 expression is associated with proliferation, increased cell survival, and disruption of cell-fate signals controlling hepatocyte metaplasia and the expansion of bipotent liver progenitor cells (LPCs) [13,14]. Nevertheless, it remains to be established whether IRS2 plays any role in hepatic wound healing.

Fibroblast growth factor 7 (FGF7) is an important paracrine regulator of tissue morphogenesis during development [15,16] and during re-epithelialization of cutaneous lesions [17]. In the liver, FGF7 is expressed by HSCs [18] and Thy1 T-cell surface antigen (Thy1)-expressing PFs [4,19] that produce stromal niche signals to drive epithelial remodeling in response to chronic injury. Fgf7-knockout mice have reduced survival due to liver failure when fed a hepatotoxic diet containing 0.1% 3.5-diethoxycarbonyl-1.4-dihydrocollidine (DDC) because they are unable to support the expansion of adult liver stem cells/LPCs required for the adaptive response to injury [19,20]. LPCs are bipotent epithelial precursors capable of differentiating into cholangiocytes or parenchymal hepatocytes [21]. During DDC liver injury, they proliferate to form duct-like structures within periportal tracts, surrounded by fibrotic stromal cells expressing Fgf7. LPCs express Fgfr2-IIIb [19], the receptor for Fgf7, which is exclusively expressed in epithelia. Amplification of Fgfr2-IIIb occurs in LPCs within the expanding nonparenchymal cell (NPC) fraction during DDC injury [22], and potentiation of Fgfr2-IIIb signaling by overexpression of Fgf7 [19] or Fgf10 [22] drives the dramatic expansion of LPCs and immature hepatocytes within the liver. Fgf7/Fgfr2-IIIb interactions therefore communicate proregenerative signals between stroma and epithelia, with a range that is strictly limited to local paracrine effects because of the high affinity of Fgf7 for heparin-sulphate proteoglycans in the extracellular matrix and neighboring cells [23].

Usually, insulin signals are systemic, but in model organisms such as *Drosophila*, they also coordinate short-range communication between niche cells and stem cells [24]. This allows for tissue-specific responses to changing environmental conditions [25]. In this study, we show that integration of insulin/IRS2 signals by HSCs and LPCs promotes paracrine crosstalk between the fibrotic stroma and LPCs via Fgf7. We demonstrate that loss of *Irs2* has a negative impact on hepatic wound healing, reducing the capacity of HSCs and LPCs to produce and respond to Fgf7 respectively.

## Results

### The response to DDC liver injury is blunted in *Irs2*^−/−^ mice

**Fig 1:**
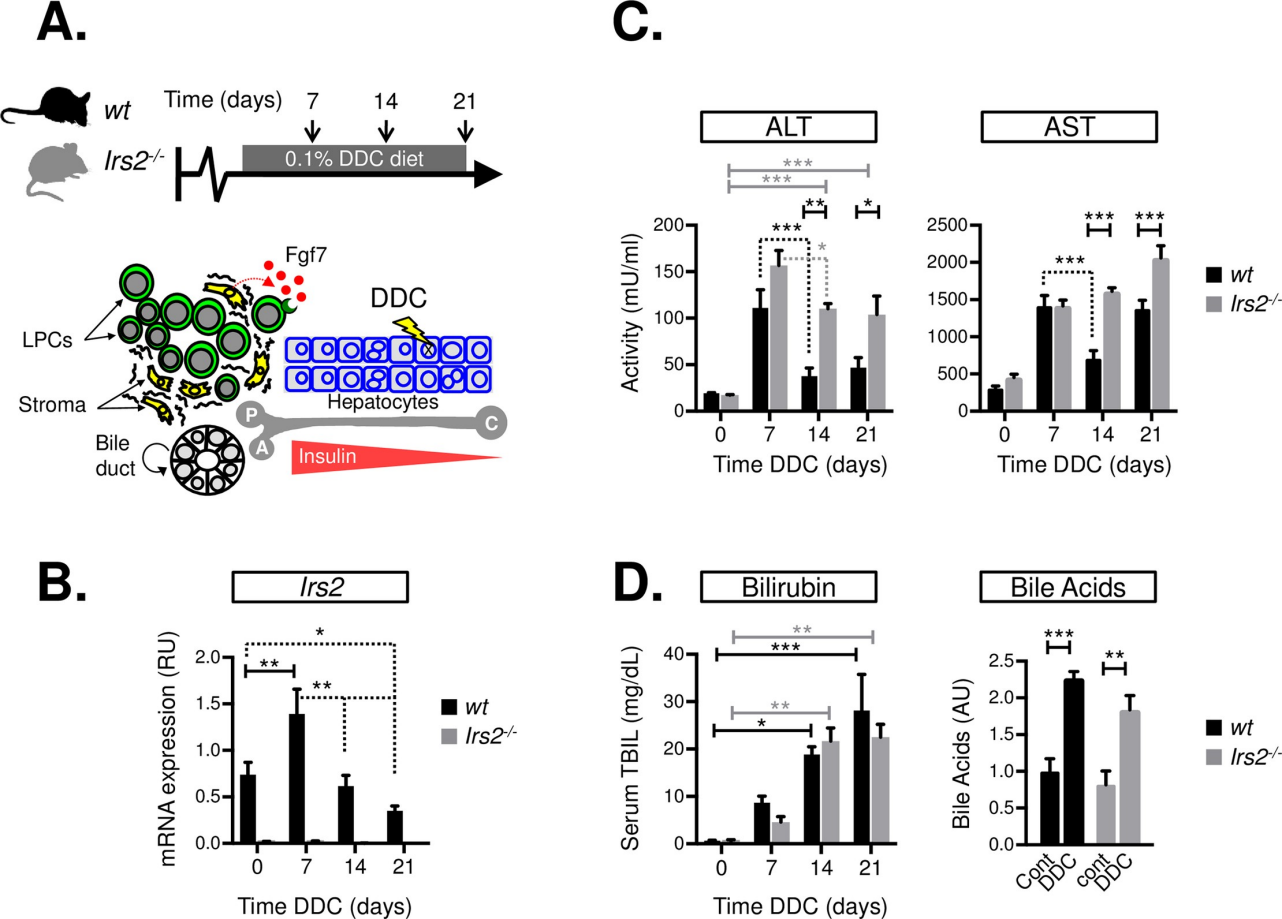
*Irs2* is required for the amelioration of liver injury during DDC feeding. (A) Schematic showing DDC feeding protocol (upper panel) and the cellular impact of chronic DDC feeding on the liver (lower panel): Hepatocellular damage is accompanied by expansion of bipotent LPCs and fibrotic stroma in periportal tracts, where cells are exposed to high concentrations of insulin. Portal vein (P), Hepatic artery (A), and Central vein (C). (B) Whole-liver mRNA levels of *Irs2* in WT and *Irs2*^−/−^ mice during a time course of DDC feeding based on RT-qPCR analysis (*n* = 3–8). Expression data are presented in RUs normalized to ribosomal protein L19 (*Rpl19*). (C) Liver injury measured by serum transaminase levels AST/ALT during a time course of DDC feeding in WT and *Irs2*^−/−^ mice (*n* = 4). (D) Equivalent levels of cholestasis in WT and *Irs2-*deficient mice during DDC injury. Left—serum levels of TBIL during a time course of DDC feeding (*n* = 3). Right—Whole-liver tissue bile acid levels prior to (Cont) and after 21 days of DDC feeding (DDC) (*n* = 4). Data information: underlying data are available in S1 Data. Values plotted as mean + SEM: Two-way ANOVA was used to compare means. Significance *P* values were calculated using Bonferroni test *P < 0.05, **P <0.01, and ***P < 0.001. Dotted lines indicate statistically significant decrease with time. ALT, alanine transaminase; AST, aspartate transaminase; Cont, control; DDC, 3.5-diethoxycarbonyl-1.4-dihydrocollidine; Fgf7, fibroblast growth factor 7; *Irs2*, insulin receptor substrate 2; LPC, liver progenitor cell; mRNA, messenger RNA; RT-qPCR, reverse transcriptase-quantitative PCR; RU, relative unit; TBIL, total bilirubin; WT, wild type.

We examined how *Irs2* deletion affected the chronic liver-injury response in adult mice during 0.1% DDC feeding, in which paracrine Fgf7 signaling plays a central role in coordinating the LPC response and epithelial repair (Fig 1A). Transient induction of *Irs2* occurred in wild-type (WT) livers on day 7 of the DDC diet (Fig 1B), correlating with a peak in liver injury as judged by serum aspartate/alanine transaminase (AST/ALT) activity (Fig 1C). No differences in serum bilirubin or liver tissue bile acid levels were observed between the two groups (Fig 1D), and initial induction of AST/ALT was comparable on day 7, suggesting the cholestatic effects of the DDC diet were equivalent in WT and *Irs2*^−/−^ mice. However, past day 7, serum transaminases were reduced in controls (days 14–21), whereas in the *Irs2*^−/−^ mice, they remained significantly elevated (Fig 1C), suggesting a role for *Irs2* in the attenuation of liver damage.

### Fgf7 expression and LPC expansion are impaired in *Irs2*^−/−^ mice

**Fig 2:**
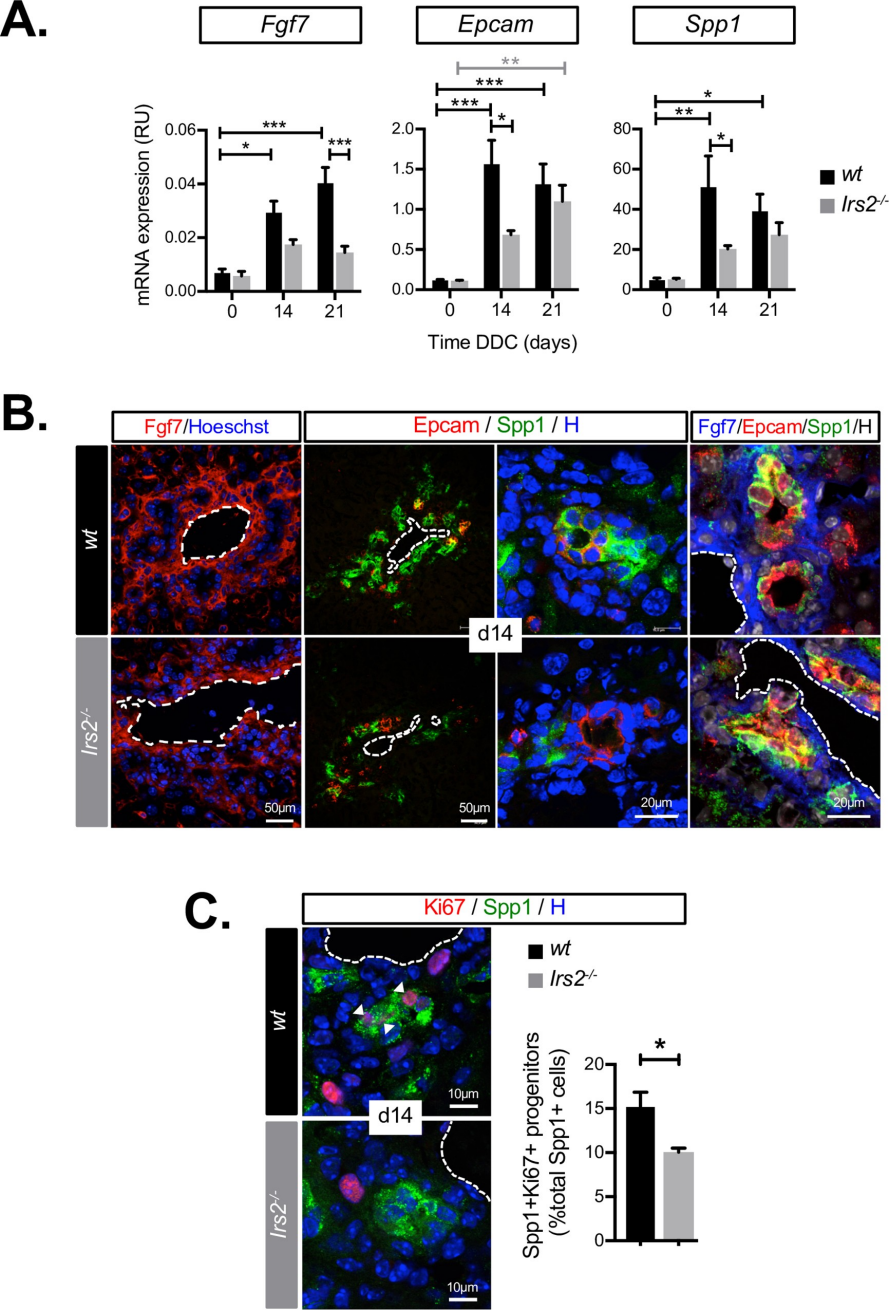
Fgf7 induction and the LPC response are impaired in *Irs2*^−/−^ mice during DDC injury. (A) RT-qPCR analysis of indicated genes in whole-liver mRNA from WT and *Irs2*^−/−^ mice during a time course of DDC feeding (*n* = 6–8). (B) Immunofluorescence staining for Fgf7-expressing stroma and EpCAM-Osteopontin/Spp1-expressing LPC ducts in livers of WT and *Irs2*^−/−^ mice after 14 days of DDC feeding. Dotted lines indicate portal vein (H = Hoechst). (C) Quantification of LPC proliferation in livers of WT and *Irs2*^−/−^ mice on DDC day 14. (left) Spp1/Ki67 immunofluorescence staining highlighting proliferation of cells within ducts (arrow heads). Dotted lines indicate portal vein. (Right) Quantification by INcell analysis of Spp1 cell proliferation (*n* = 6, total of 6.47 × 10^4^ Spp+ cells analyzed). Data information: underlying data are available in S1 Data. Values plotted as mean + SEM. *P < 0.05, **P < 0.01, and ***P < 0.001. (A) Two-way ANOVA was used to compare means. Significance *P* values were calculated using Tukey's multiple comparison test. (C) Unpaired Student *t* test. DDC, 3.5-diethoxycarbonyl-1.4-dihydrocollidine; EpCAM, epithelial cell adhesion molecule; Fgf7, fibroblast growth factor 7; *Irs2*, insulin receptor substrate 2; LPC, liver progenitor cell; mRNA, messenger RNA; RT-qPCR, reverse transcriptase-quantitative PCR; RU, relative unit; Spp1, secreted phosphoprotein 1; WT, wild type.

We observed robust Fgf7 activation in WT livers (days 14–21), which coincided with the significant reductions in AST/ALT recorded at these time points (Figs 2A and 1B), consistent with the proregenerative role played by Fgf7 in the response to DDC injury. Fgf7-expressing stromal cells surrounded duct-like structures in periportal tracts that stained positive for LPC markers including epithelial cell adhesion molecule (EpCAM) and osteopontin (secreted phosphoprotein 1, Spp1) (Fig 2B). In *Irs2*^−/−^ mice, we observed a striking failure to induce Fgf7 during DDC feeding (Fig 2A). We also observed modest but significant down-regulation of *Fgf10* but no change in *Fgf22* (S1 Fig), suggesting other stromal Fgfr2-IIIb ligands were also affected. Failure to induce *Fgf7* expression in *Irs2*^−/−^ livers occurred in parallel with a delay in the overall increase in hepatocyte nuclear factor 4-alpha (HNF4α)− NPCs (S2A and S2B Fig), as well as a specific delay in the induction of LPC genes *EpCAM* and *osteopontin*/*Spp1* (Fig 2A) and decreased ductular immunostaining in periportal areas (Fig 2B). Proliferative expansion of cells expressing osteopontin/Spp1 was also diminished (day 14), as judged by Ki67 immunostaining (Fig 2C), and ducts containing LPCs appeared disorganized (Fig 2B).

Parenchymal cell depletion was exacerbated in *Irs2*^−/−^ mice during DDC feeding, based on quantification of HNF4α+ hepatocyte nuclei (S2A and S2C Fig). This was characterized by failure to sustain numbers of so-called "small" hepatocytes (nuclear area < 75 μm^2^), parenchymal cells attributed with the greatest regenerative potential (S2D Fig) [26,27]. Using an original methodology to estimate ploidy (described in Materials and methods), we analyzed changes in hepatocyte DNA content in whole-liver tissue sections during DDC injury (S3A and S3B Fig). We found that the population of small hepatocytes with approximately 2n DNA content (2c) was increased in the WT group during the later stages of DDC injury (days 14–21, 1.6-fold *P* = 0.0335), consistent with a regenerative response (S3C Fig). During the same period, 2c hepatocytes declined in numbers in the livers of *Irs2*^−/−^ mice, indicating a failure to maintain parenchymal tissue homeostasis during chronic injury.

### Altered profile of stromal activation in *Irs2*^−/−^ mice highlights aberrant response by Fgf7-expressing mesenchymal cells

**Fig 3:**
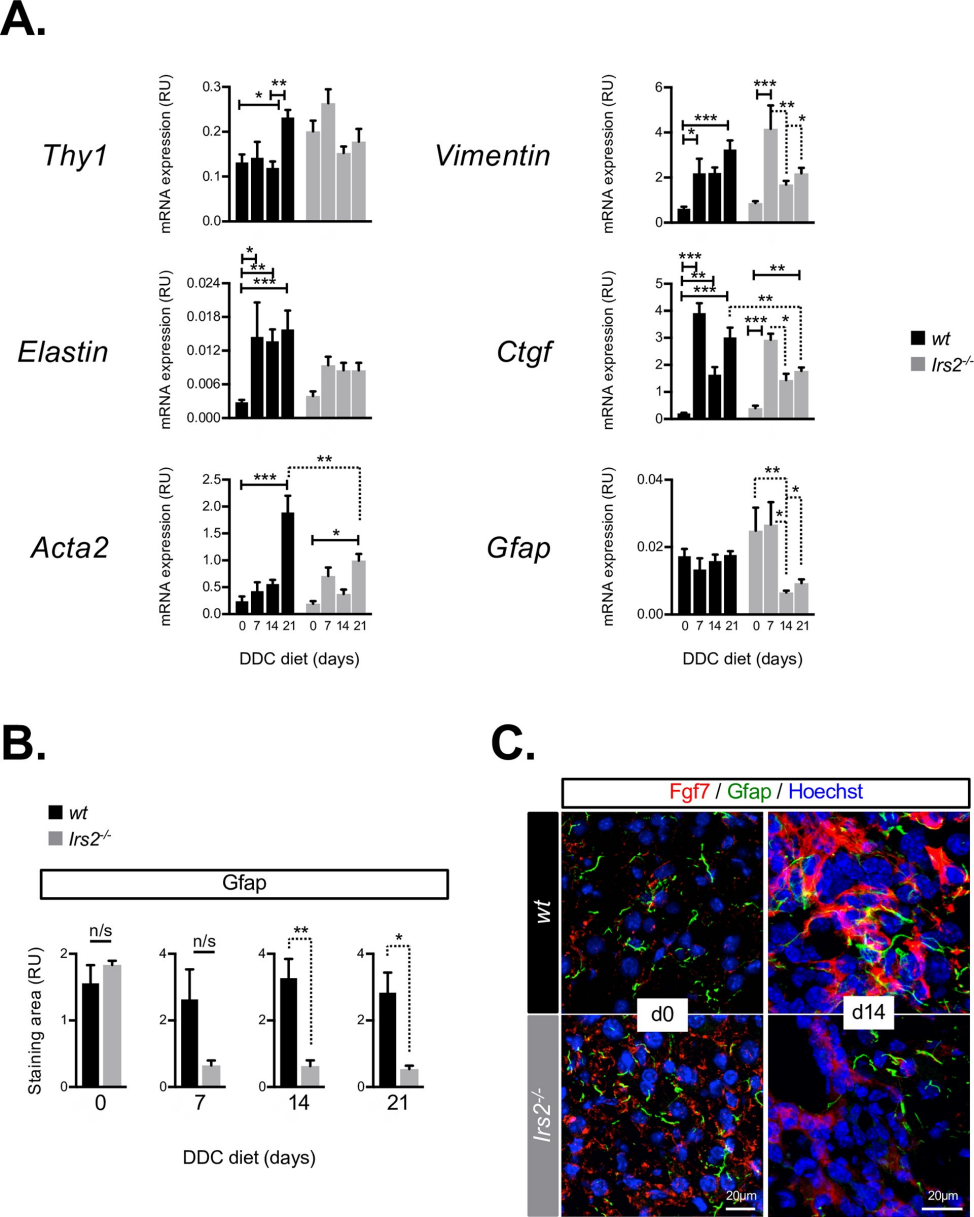
The stromal response to DDC injury in *Irs2*^−/−^ mice is characterized by loss of HSCs and blunted activation of PF/myofibroblast gene expression. (A) RT-qPCR analysis of whole-liver mRNA during a time course of DDC injury using a panel of genes associated with Fgf7-expressing stromal cells: PFs (*Thy1*, *Elastin*), myofibroblasts (*Acta2*, *Vimentin*, *Ctgf*), and HSCs (*Gfap*). In livers of *Irs2*^−/−^ mice, we observed impaired or less sustained induction of PF/myofibroblast markers (*Thy1*, *Elastin*, *Acta1*) combined with a tendency for all genes (except *Elastin*) to be down-regulated from day 7 in parallel with loss of *Gfap* (*n* = 3–8). (B) Gfap+ HSCs were depleted in *Irs2*^−/−^ mice during DDC injury. Quantification of Gfap immunostaining in the livers of WT and *Irs2*^−/−^ mice during the time course of DDC injury (*n* = 3–4). (C) Loss of Gfap+ HSCs in *Irs2*^−/−^ mice parallels the failure to induce Fgf7 in periportal areas. Confocal immunofluorescence images showing reduced Gfap/Fgf7 double staining in *Irs2*^−/−^ mice after 14 days of DDC feeding (day 14). Data information: underlying data are available in S1 Data. Data are presented as mean + SEM. *P < 0.05, **P < 0.01, and ***P < 0.001. (A) Two-way ANOVA was used to compare means. Significance *P* values were calculated using Tukey's multiple comparison test. (B) Unpaired Student *t* test. *Acta1*, alpha-smooth muscle actin; *Ctgf*, Connective tissue growth factor; DDC, 3.5-diethoxycarbonyl-1.4-dihydrocollidine; Fgf7, fibroblast growth factor 7; Gfap, glial fibrillary acidic protein; HSC, hepatic stellate cell; *Irs2*, insulin receptor substrate 2; mRNA, messenger RNA; PF, portal fibroblast; RT-qPCR, reverse transcriptase-quantitative PCR; RU, relative unit; WT, wild type.

In rodent and human livers, Fgf7 is expressed by HSCs during injury [18]. However, in the DDC mouse model, resident Thy1+ PFs have also been identified as important Fgf7-expressing mesenchymal cells and first responders to cholestatic liver damage [4,19]. Profiling of stromal gene expression in WT mice during DDC injury revealed sustained induction of PF/myofibroblast genes *Thy1*, *Elastin*, *Vimentin*, and alpha-smooth muscle actin (αSma*/Acta2*), together with profibrogenic gene Connective tissue growth factor (*Ctgf*) (Fig 3A), whereas in *Irs2*^−/−^ mice, the activation profile of these genes was either blunted (*Thy1*, *Elastin*), less sustained (*Acta2*), or down-regulated from day 7 (*Vimentin*, *Ctgf*, and *Thy1*), suggesting a negative impact on the PF/myofibroblast population within the activated stroma. Interestingly, we also observed a decline in expression of the HSC marker glial fibrillary acidic protein (*Gfap*) from day 7 (Fig 3A), coincident with significant reductions in mesenchymal marker *Vimentin* and in *Ctgf*, a regulator of epithelial mesenchymal transition. From day 7, a significant decrease in Gfap immunostaining in *Irs2*^−/−^ livers was also observed (Fig 3B and 3C). This reduction in Gfap+ cells was apparent throughout the parenchyma and in periportal areas where it colocalized with Fgf7-expressing stroma (Fig 3C).

In contrast with the results of messenger RNA (mRNA) analysis (Fig 3A), immunostaining for Thy1, Elastin, and αSMA tended towards increased expression in *Irs2*^−/−^ livers on day 21, while mesenchymal intermediate filament protein vimentin was similarly induced (S4 Fig). Thy1+ stroma were observed in close contact with Spp1+/Epcam+ LPCs in both WT and *Irs2*^−/−^ mice as previously described [19] (S5A Fig), and a similar relationship between αSma-expressing myofibroblasts and Spp1+ LPCs was also apparent in both groups (S5B Fig), indicating that failure to induce Fgf7 was not due to the lack of myofibroblast-like cells in the LPC niche of *Irs2*^−/−^ livers per se. In contrast, lack of contact between Gfap+ HSCs and LPCs was observed in *Irs2*^−/−^ livers (S5B Fig). Hence, failure to induce Fgf7 coincided with an overall loss of Gfap+ HSCs and loss of association between HSCs and LPCs in portal tracts, whereas we were unable to confirm that stromal PFs were negatively influenced by *Irs2* deletion. However, increased Thy1 staining in *Irs2*^−/−^ livers was partially attributable to the increase in markers of bone-marrow–derived cells; cd45/protein tyrosine phosphatase receptor type C gene encoding CD45 cell surface antigen (*Ptprc*) and cd117/Proto-oncogene c-kit (*Kit*), on day 7, which coincided with early activation of matrix remodeling factors (tissue inhibitor of metalloproteinase 1 [*Timp1*] and Matrix metallopeptidase 9 [*Mmp9*]) and MYC proto-oncogene (*cMyc*) and a dramatic early peak in transforming growth factor beta (*Tgf*β)—a master regulator of fibrogenesis and mobilizer of circulating populations of collagen-expressing fibrocytes [28] (S6A Fig). Consistent with this, we also observed greater Thy1/Cd45 colocalization in *Irs2*^−/−^ livers on day 21 (S6B Fig), suggesting more extensive incorporation of bone-marrow–derived cells into the stromal niche. Furthermore, the numbers of Cd3+ T cells were also increased in *Irs2*^−/−^ livers on day 21 (S6C Fig), suggesting that Thy1+ cells from the bone marrow, whose expression of Fgf7 is lower than that of resident Thy1+ fibroblast populations [4], played a more significant role in the altered stromal response to DDC injury in *Irs2*^−/−^ mice.

### Survival of Fgf7-expressing HSCs is *Irs2* dependent

**Fig 4:**
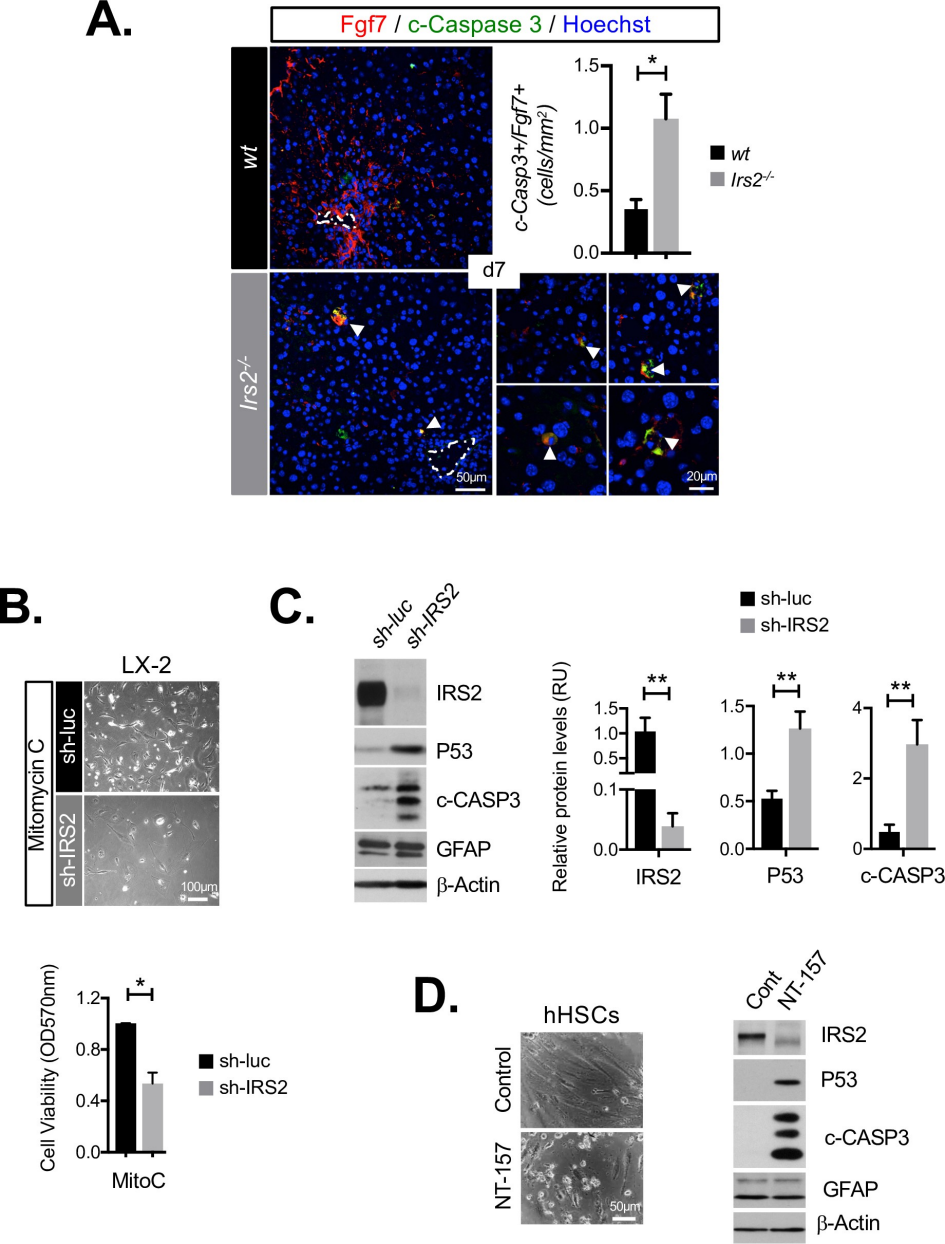
*Irs2* depletion leads to reduced survival of Fgf7-expressing stroma and human HSCs. (A) Representative immunofluorescence images and graphical quantification of cleaved caspase 3 staining in Fgf7-expressing stroma in livers of WT and *Irs2*^−/−^ mice after 7 days of DDC feeding (*n* = 3), duplicates analyzed for each animal, mean total area 33.9 mm^2^ per section. Dotted lines indicate portal vein. (B–C) IRS2 protects hHSC-derived LX-2 cells from apoptotic cell death following MitoC treatment in vitro. LX-2 cells were stably transduced with sh-luc or sh-IRS2 and treated with MitoC. (C) Representative phase-contrast images showing reduced survival of sh-IRS2 cells at 72 h post-MitoC treatment (above). Graph below shows results of MTT cell-viability analysis, also performed at 72 h, confirming reduced survival (*n* = 3). (D) Western blot analysis with graphical quantitation of indicated relative protein expression levels (*n* = 3–5). (D) Inhibition of IRS proteins in primary hHSCs activates apoptotic cell death. Cells were treated with NT-157 (5 μM) or Control (DMSO) for 72 h. Phase-contrast images showing dramatic cell death induced by NT-157 treatment (left) and western blot (representative of *n* = 4) showing cleaved caspase and P53 activation corresponded with reduced IRS2 levels. Data information: underlying data are available in S1 Data. Data are presented as mean + SEM. *P < 0.05, **P < 0.01, and ***P < 0.001. (A) Unpaired Student *t* test. (B–C) Paired Student *t* test. c-CASP3, cleaved caspase 3; Cont, control; Fgf7, fibroblast growth factor 7; Gfap, glial fibrillary acidic protein; hHSC, human HSC; HSC, hepatic stellate cell; *Irs2*, insulin receptor substrate 2; MitoC, mitomycin C; MTT, 3-(4,5-dimethylthiazol-2-yl)-2,5-diphenyltetrazolium bromide; NT-157, allosteric inhibitor of IRS1 and 2; RU, relative unit; sh-IRS2, shRNA-targeting IRS2; sh-luc, control luciferase; shRNA, short hairpin RNA; WT, wild type.

We assessed the survival of Fgf7-expressing stroma during the early phase of DDC injury, when *Irs2* was maximally expressed in WT livers (day 7) and before significant loss of Gfap+ HSCs was observed. Analysis of cleaved caspase 3 (c-Casp3) staining revealed a 3.0-fold (*P* = 0.0395) increase in apoptosis in Fgf7-expressing cells in *Irs2*^−/−^ livers at this time, confirming accelerated loss of this subpopulation within the fibrotic stroma during injury (Fig 4A). In order to test the hypothesis that *IRS2* promoted survival of Fgf7-expressing stroma, we performed stable knockdown of *IRS2* in the human HSC (hHSC) cell line LX-2 using lentiviral short hairpin RNA (shRNA) (Fig 4B and 4C). Silencing of *IRS2* had no direct impact on LX-2 cell viability, Fgf7 expression, or fibrogenic gene expression under standard culture conditions (S7 Fig). However, exposure to cytotoxic alkylating agent mitomycin C (MitoC) resulted in cell-cycle arrest and apoptotic cell death, during which *IRS2* promoted survival (Fig 4B) by significantly reducing P53 expression and caspase 3 cleavage (Fig 4C). We confirmed a positive role for *IRS2* in protecting HSCs from apoptosis by treating primary hHSC cultures with an allosteric inhibitor of IRS proteins (NT-157). NT-157 triggers proteasomal depletion of IRS1/IRS2 [29], which resulted in dramatic activation of P53/caspase 3 cleavage and apoptosis in hHSCs (Fig 4D). We therefore concluded that IRS2 plays an indirect role in driving Fgf7 expression during DDC injury by protecting HSCs from apoptosis.

### *Irs2* promotes epithelial sensitivity to Fgf7 and hepatic re-epithelialization during injury

**Fig 5:**
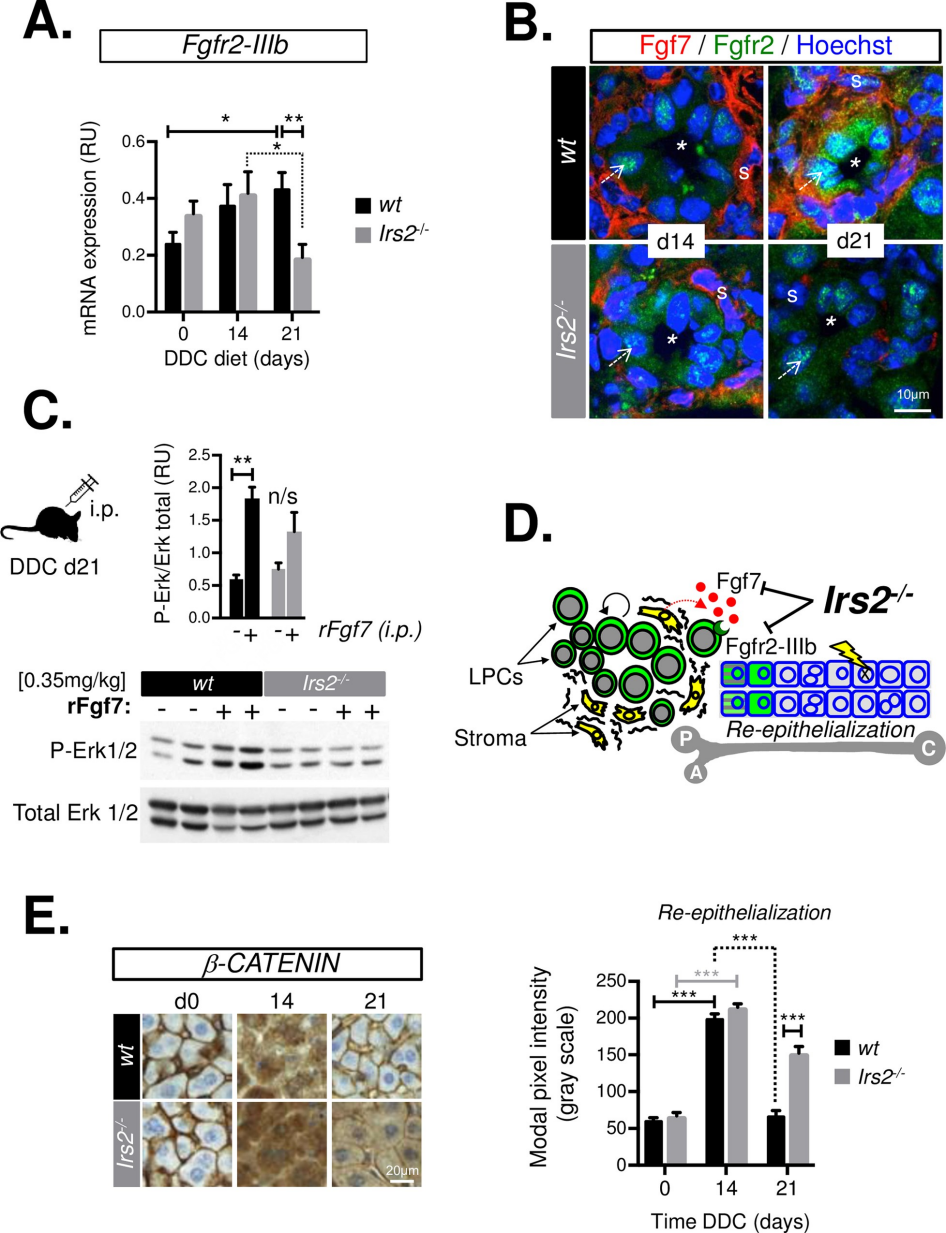
*Irs2* is required to sustain hepatic *Fgfr2-IIIb* expression and re-epithelialization during DDC injury. (A) RT-qPCR analysis of *Fgfr2-IIIb* expression in whole-liver mRNA from WT and *Irs2*^−/−^ mice during a time course of DDC feeding (*n* = 6–8). (B) Immunofluorescence images of WT and *Irs2*^−/−^ livers after 14 and 21 days of DDC feeding. Induction of Fgfr2 occurred within duct-like structures in periportal areas (*) surrounded by Fgf7-expressing stroma (s). The failure to sustain Fgf7 expression in Irs2^−/−^ mice was paralleled by concomitant failure to induce Fgfr2 in LPCs (arrows). (C) The livers of Irs2^−/−^ mice are refractory to exogenous Fgf7 stimulation following DDC injury. Western blot analysis of whole-liver extracts from DDC-treated WT and *Irs2*^−/−^ mice (day 21). 30 minutes prior to being killed, mice were administered rFgf7 or vehicle by i.p. injection. P-Erk levels relative to total Erk were quantified and are shown in the graph above (*n* = 4–7), and representative images of blots are shown below. (D) Schematic summarizing the observed negative impact of *Irs2* deletion on Fgf7 signaling between the hepatic stroma and LPCs during DDC injury. P = portal vein, A = hepatic artery, C = central vein. (E) Immunohistochemical staining for β-catenin in WT and *Irs2*^−/−^ liver parenchyma during a time course of DDC injury. Modal pixel intensity was graphed and used to quantify the dramatic remodeling of β-catenin staining (days 0–14) and reestablishment of epithelial architecture in livers of WT but not *Irs2*^−/−^ mice in the latter period of the diet (days 14–21) (*n* = 6–9). Data information: underlying data are available in S1 Data. Data are presented as mean + SEM. *P < 0.05, **P < 0.01, and ***P < 0.001. (A and E) Two-way ANOVA was used to compare means. Significance *P* values were calculated using Bonferroni test. (C) One-way ANOVA was used to compare means. Significance *P* values were calculated using Tukey’s test. DDC, 3.5-diethoxycarbonyl-1.4-dihydrocollidine; Erk, extracellular signal-regulated kinase; Fgfr2-IIIb, Fgf7 ligand and receptor; Fgf7, fibroblast growth factor 7; i.p., intraperitoneal; *Irs2*, insulin receptor substrate 2; LPC, liver progenitor cell; mRNA, messenger RNA; P-Erk, phosphorylated Erk (Tyrosine 204); RT-qPCR, reverse transcriptase-quantitative PCR; RU, relative unit; WT, wild type.

In addition to the defect in stromal Fgf7 expression, *Irs2* deletion also had a negative impact on sensitivity to Fgf7 in epithelial cells. Stromal Fgf7 drives re-epithelialization during skin wound healing, signaling exclusively to cells expressing the epithelial isoform of Fgfr2 (Fgfr2-IIIb), which is highly expressed by LPCs during DDC feeding [19]. In *Irs2*^−/−^ livers, we noted a sharp decline of *Fgfr2-IIIb* expression in the latter stages of DDC feeding (days 14–21), which contrasted with the steady increase in *Fgfr2-IIIb* observed in the WT group (Fig 5A). In WT mice, we observed increased antibody staining for Fgfr2 in periportal ducts surrounded by Fgf7+ stroma (Fig 5B). No such induction was observed in *Irs2*^−/−^ livers, consistent with a failure of *FGFR2-IIIb* expression in LPCs. We confirmed that diminished *Fgfr2-IIIb* expression resulted in reduced Fgf7-sensitivity in injured *Irs2*^−/−^ livers by testing the signaling response to intraperitoneal (i.p.) injection of recombinant Fgf7 (rFgf7) protein. In WT mice, rFgf7 robustly activated Fgf7/Fgfr2 signaling as assessed by western blot, demonstrating increased in vivo phosphorylation of extracellular signal-regulated kinases (ERK)1/2 in whole-liver lysates (Fig 5C). This response to rFgf7 was abrogated in the *Irs2*^−/−^ livers, indicating that they were refractory to Fgf7. Taken together, our findings suggested that *Irs2* played a dual role in (i) promoting stromal expression of Fgf7 and (ii) sustaining sensitivity of the hepatic epithelia to Fgf7 during chronic DDC liver injury by promoting Fgfr2-IIIb expression (Fig 5D). Interestingly, the timing of *Fgfr2-IIIb* loss in *Irs2*^−/−^ mice (days 14–21) corresponded with a period during which the parenchymal architecture failed to re-epithelialize, as judged by quantitative analysis of β-catenin immunostaining in hepatocytes (Fig 5E), suggesting that in the later stages of DDC injury, *Irs2* was required upstream of Fgf7–Fgfr2-IIIb signaling to driving adaptive epithelial remodeling.

### Insulin/IRS2 signaling and FGF7 synergize to promote FGFR2-IIIb expression and the emergence of hepatocytic epithelia in bipotent human HepaRG cells

**Fig 6:**
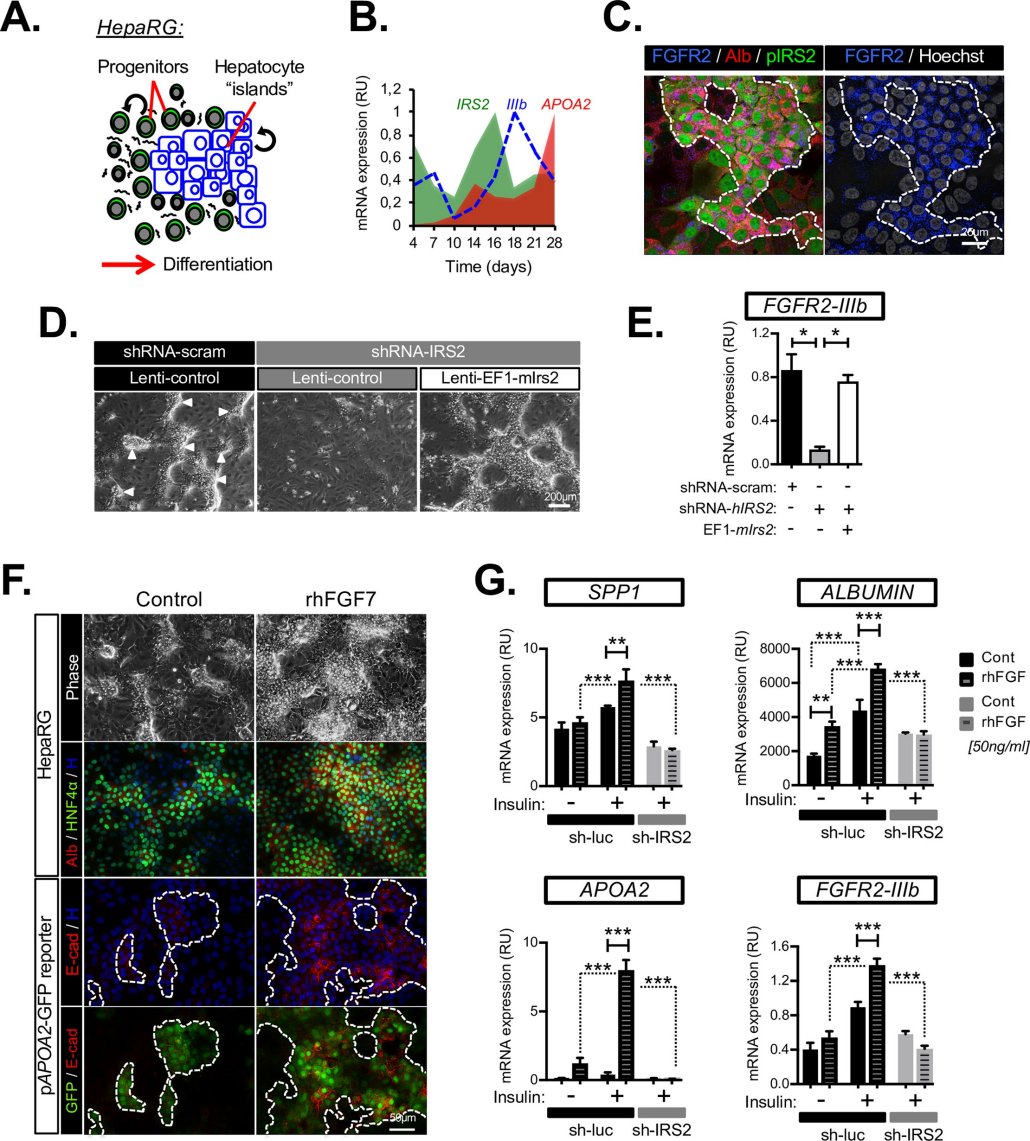
Insulin/*IRS2* signaling promotes epithelialization and sensitivity to FGF7 in vitro. (A) Schematic showing differentiation of bipotent HepaRG human LPCs in vitro produces epithelial “islands” containing hepatocyte-like cells. (B) RT-qPCR time course of HepaRG differentiation showing early induction of *IRS2*, and *FGFR2-IIIb* prior to hepatocyte differentiation (indicated by *APOA2*). (C) Colocalization of Alb, FGFR2, and IRS2 by immunostaining within hepatocyte islands during the early stages of HepaRG differentiation (day 14). *IRS2* expression was visualized by stable transduction with a human IRS2 promoter (pIRS2) GFP reporter. Dotted line delimits “island.” (D–E) *IRS2* is necessary for *FGFR2-IIIb* expression during HepaRG differentiation: (D) phase-contrast images of differentiated cells after stable silencing for *IRS2* using shRNA lentivirus (sh-IRS2). Arrowheads highlight “islands” observed in control cultures expressing sh-scram but not in sh-IRS2 cells. Specificity of the knockdown phenotype was confirmed by a “rescue” in which Lenti-mIrs2 was constitutively expressed in sh-IRS2 cells. (E) RT-qPCR analysis of FGFR2-IIIb expression (*n* = 3). (F–G) Insulin/IRS2 is required for functional sensitivity to rFGF7 in HepaRG cells and is necessary for feed-forward induction of FGFR2-IIIb by FGF7 ligand. (F) Phase-contrast and immunofluorescence images of HepaRG cells (day 15) cultured with rhFGF7 for 11 days, showing an increase in epithelial islands positive for Alb/HNF4α and E-cad immunostaining and *pAPOA2*-GFP reporter expression (H = Hoechst). (G) RT-qPCR analysis for indicated genes following long-term (11-day) rhFGF7 treatment in the presence (+) or absence (−) of supplemented insulin in control (sh-luc) or IRS2 knockdown cells (sh-IRS2) (*n* = 3). Data information: underlying data are available in S1 Data. Data are presented as mean + SEM. *P < 0.05, **P < 0.01, and ***P < 0.001. (E and G) One-way ANOVA was used to compare means. Significance *P* values were calculated using Newman–Keuls multiple comparison test. Alb, albumin; *APOA2*, apolipoprotein A2; Cont, control; E-cad, E-cadherin; EF1, Human elongation factor-1 alpha promoter; FGF7, fibroblast growth factor 7; FGFR2, fibroblast growth factor receptor 2; GFP, green fluorescent protein; HNF4α, hepatocyte nuclear factor 4-alpha; *IRS2*, insulin receptor substrate 2; Lenti-mIrs2, murine *Irs2* transgene not targeted by human-gene–silencing construct; mRNA, messenger RNA; *pAPOA2*, human *APOA2* promoter; *pIRS2*, *IRS2* promoter; rFgf7, recombinant Fgf7; rhFGF7, recombinant human FGF7; RT-qPCR, reverse transcriptase-quantitative PCR; RU, relative unit; sh-IRS2, shRNA-targeting IRS2; sh-luc, control luciferase; shRNA, short hairpin RNA; sh-scram, scrambled shRNA.

We examined the possibility that IRS2 played a direct role in regulating LPC sensitivity to FGF7 and epithelialization using a human model of bipotent adult liver progenitor cells (HepaRG) [30]. Upon reaching confluence, HepaRG cells spontaneously differentiate in media supplemented with insulin to produce phase-bright epithelial “islands” of hepatocyte-like cells (Fig 6A and S8 Fig). Within these “islands,” cells express hepatocyte genes such as albumin, *HNF4α*, cytochrome P450 3A4 (*CYP3A4*), and apolipoprotein A2 (*APOA2*) (S8 Fig). Using this model, we found that *IRS2* was induced prior to *FGFR2-IIIb* and hepatocyte-specific genes such as *APOA2* (Fig 6B). Using a human *IRS2*-promoter construct to visualize the pattern of *IRS2* expression via green fluorescent protein (p*IRS2*-GFP), we also demonstrated colocalization of *IRS2* with albumin and FGFR2 in the early stages of hepatocyte island formation (Fig 6C). These data were consistent with an early role for IRS2 in the patterning of FGF7 sensitivity during LPC differentiation.

Removal of insulin from the media (S8B and S8C Fig) or stable silencing of *IRS2* (Fig 6D, S8D–S8F Fig) resulted in a striking failure to generate hepatocytic epithelia within the cultures, together with concomitant loss of *FGFR2-IIIb* expression (Fig 6E). Importantly, the formation of epithelial islands and *FGFR2-IIIb* were restored by exogenous expression of a mouse *Irs2* not targeted by the human *IRS2* shRNA construct (Fig 6D and 6E); hence, we confirmed a cell-autonomous role for *IRS2* in promoting FGFR2-IIIb expression and differentiation in LPC-like cells.

Culturing of HepaRG cells with recombinant human FGF7 (rhFGF7) accelerated the formation of islands and increased hepatocyte differentiation, as determined by increased albumin/HNF4α and E-cadherin (E-cad) immunostaining, human *APOA2*-promoter (*pAPOA2*)-GFP reporter activity (Fig 6F), and analysis of hepatocyte-specific gene expression (Fig 6G). This occurred downstream of a rapid (3–6 h) and sustained induction in osteopontin/*SPP1* (S9 Fig), consistent with our in vivo observation that osteopontin/*Spp1* and *Fgf7* were both impaired in *Irs2*^−/−^ livers during injury (Fig 2). Importantly, the ability of rhFGF7 to promote expression of osteopontin/*SPP1*, as well as hepatocyte-specific genes such as albumin and *APOA2*, was both insulin and *IRS2* dependent (Fig 6G), revealing synergy between these two pathways in driving the differentiation of bipotent progenitor cells. Stable silencing of *IRS2* or omission of supplemented insulin from the medium was sufficient to abrogate the positive long-term effects of rhFGF7 on the hepatogenic program, thus demonstrating a functional requirement for insulin/IRS2 upstream of paracrine FGF7 signaling. Interestingly, rhFGF7 treatment also increased *FGFR2-IIIb* expression, highlighting the potential for positive feedback between ligand and receptor—an effect that was also insulin/IRS2 dependent (Fig 6G) and hence reinforced the conclusion that insulin/IRS2 signaling in LPC-like cells primed FGF7 sensitivity.

Our data supported the hypothesis that insulin/IRS2 signaling was functionally upstream of FGF7-mediated epithelialization of LPCs and highlighted a cell-intrinsic role for *IRS2* in promoting sensitivity to paracrine FGF7 produced by the fibrotic stroma. However, they also revealed that loss of stromal FGF7 could by itself be sufficient to explain a decline in *FGFR2-IIIb* expression and epithelialization in a non-cell–autonomous manner as a consequence of reduced positive feedback between ligand and receptor.

### Insulin/IRS2 resistance in fibrotic stroma limits hepatocyte differentiation and *FGFR2-IIIb* expression in cocultured LPCs

**Fig 7:**
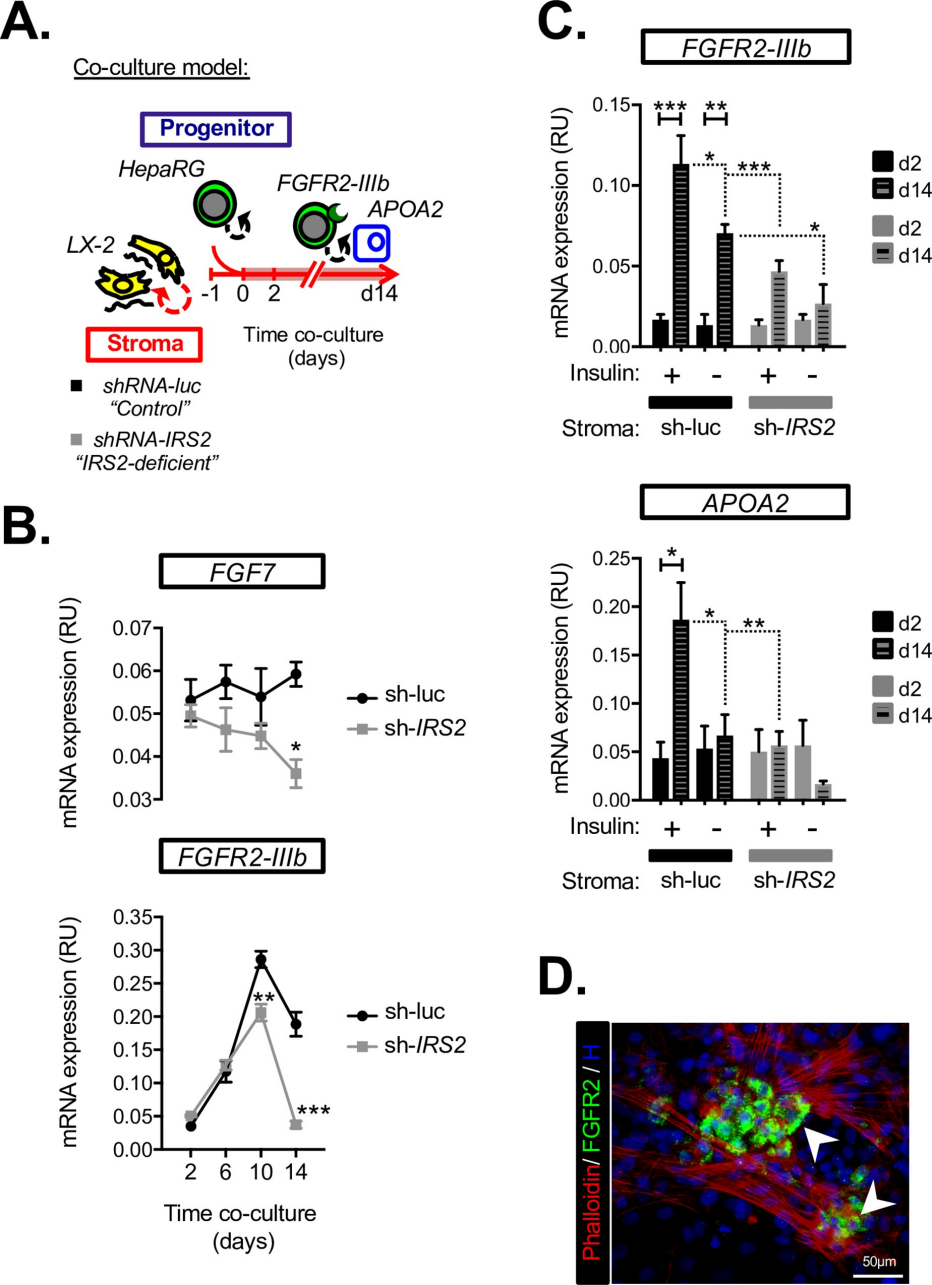
Heterotypic cell–cell crosstalk between HSCs and LPCs is IRS2 dependent. (A) Schematic illustrating the HSC/LPC coculture model designed to test whether *IRS2* expression in the stromal cell compartment was required for heterotypic interactions leading to increased *FGFR2-IIIb* expression and APOA2 induction in adjacent HepaRG cells. Control (shRNA-luc) or IRS2-deficient (shRNA-IRS2) LX-2 cells were seeded for a day prior to addition of HepaRG cells as indicated, and the resulting cocultures were then studied for 2–14 days. (B) RT-qPCR time course of gene expression in the HSC/LPC coculture system showing stromal IRS2 expression was required for sustained *FGF7* expression and induction of *FGFR2-IIIb* (*n* = 3). (C) RT-qPCR analysis of control (sh-luc) and IRS2-deficient (sh-IRS2) LX-2 cocultures performed in the presence (+) or absence (−) of supplemented insulin (*n* = 3). (D) Coculture immunostaining (day 14) highlighting localized FGFR2 expression (arrows) at sites of contact between HepaRG and HSC stroma (marked by phalloidin). Data information: underlying data are available in S1 Data. Data are presented as mean + SEM. *P < 0.05, **P < 0.01, and ***P < 0.001. (B) Two-way ANOVA was used to compare means. Significance *P* values were calculated using Tukey's multiple comparison test. (C) Two-way ANOVA was used to compare means. Significance *P* values were calculated using Sidak's multiple comparison test. *APOA2*, apolipoprotein A2; Fgfr2-IIIb, Fgf7 receptor; Fgf7, fibroblast growth factor 7; H, Hoechst; HSC, hepatic stellate cell; *Irs2*, insulin receptor substrate 2; LPC, liver progenitor cell; mRNA, messenger RNA; RT-qPCR, reverse transcriptase-quantitative PCR; RU, relative unit; shIRS2, shRNA-targeting IRS2; sh-luc, control luciferase; shRNA, short hairpin RNA.

Our results highlighted the potential for a paracrine feed-forward loop in which FGF7 drove LPC differentiation and *FGFR2-IIIb* expression, potentiating both epithelialization and FGF7 sensitivity in an insulin/IRS2-dependent manner. To test this hypothesis further and to more rigorously address the role of insulin/IRS2 signaling in the fibrotic stroma, we performed coculture experiments with hHSCs and HepaRG cells, seeding the two cell types together in order to model the intimate heterotypic cell–cell interactions observed between LPCs and their stromal niche in periportal tracts during DDC injury (Fig 7A). LX-2 control (sh-luc) and *IRS2*-knockdown (sh-*IRS2*) cells were used to simulate "normal" versus "*IRS2*-deficient" fibrotic stroma and gauge how stromal insulin/IGF-1 resistance influenced the LPC response in a non-cell–autonomous manner.

Consistent with the hypothesis that *FGF7* was indirectly regulated by stromal IRS2, we observed similar levels of *FGF7* in the early stages of LX-2/HepaRG coculture (Fig 7B). However, while *FGF7* expression was sustained in cocultures containing normal LX-2 stroma, a significant long-term decline was observed in those that contained *IRS2*-deficient LX-2 (Fig 7B), demonstrating an indirect role for stromal IRS2 in promoting *FGF7* expression in coculture. The decline *FGF7* expression in the *IRS2*-deficient stromal cocultures also coincided with a striking failure to induce *FGFR2-IIIb* on day 14 (Fig 7B). Interestingly, we also observed blunted induction of *FGF10* within cocultures containing *IRS2*-deficient stroma (days 10–14), whereas *FGF22* expression was unaffected (S10A Fig). These data reinforced the notion that FGFR2-IIIb ligands such as FGF7/FGF10 were required to drive expression of their epithelial receptor in LPCs both in vivo and in vitro.

Using the coculture method, we also found that *FGFR2-IIIb* and *APOA2* induction by LPCs required insulin signaling via IRS2 within the fibrotic stroma. Knockdown of *IRS2* in LX-2 cells completely abrogated the ability of insulin to potentiate epithelial sensitivity to FGF7 and hepatocyte differentiation within the cocultures, as judged by loss of time-dependent induction of *FGFR2-IIIb* and *APOA2*, respectively (Fig 7C). These results demonstrated a requirement for stromal *IRS2* in the non-cell–autonomous potentiation of FGF7 target genes in HepaRG cells. Moreover, we observed localized expression of FGFR2 in HepaRG at sites of contact with LX-2 cells, suggesting regulation of LPC differentiation by the stroma at close range, consistent with the short-rage paracrine actions of FGF7 (Fig 7D).

**Fig 8:**
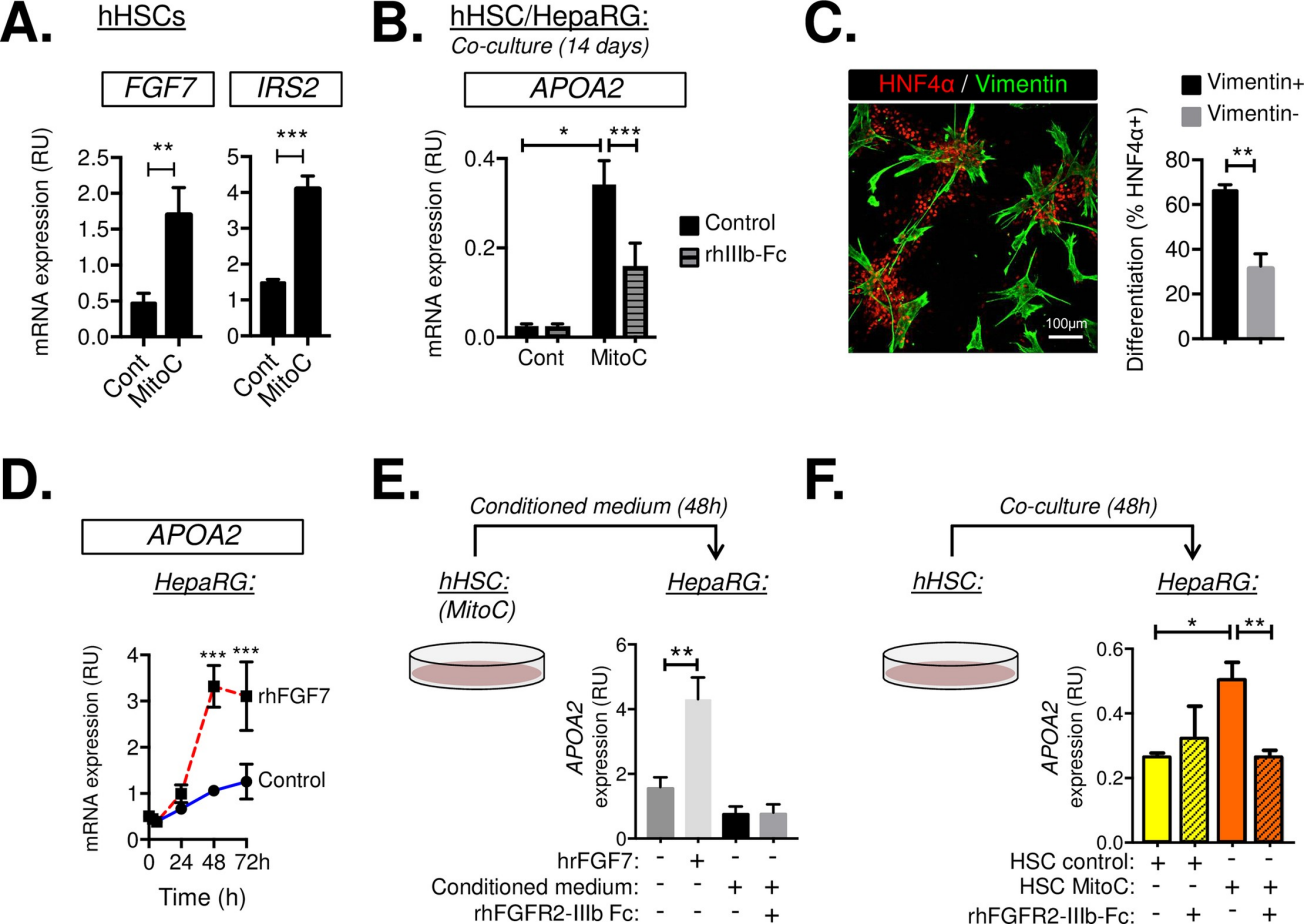
Paracrine FGF7 signaling by HSCs is activated during cell-cycle arrest. (A) RT-qPCR analysis of primary hHSC cultures treated with vehicle (cont) or MitoC 72 h prior to harvesting. *FGF7* and *IRS2* expression were enhanced by MitoC (*n* = 3). (B) MitoC treatment of hHSCs increased paracrine FGF7 signaling. Cont/MitoC-treated hHSCs were cocultured with HepaRG cells for 14 days in the presence/absence of the rhFGFR2-IIIb-Fc fusion protein (rhIIIb-Fc) used to block Fgf7 binding its receptor. Cocultures were then analyzed by RT-qPCR for *APOA2* expression (*n* = 3) as a functional readout of hepatocyte differentiation and FGF7 target gene activity. (C) HNF4α/Vimentin immunostaining of MitoC-treated hHSC/HepaRG cocultures (day 14). HepaRG differentiation (HNF4α+) was highly localized to sites of contact between hHSCs (Vimentin+). Graph shows INcell analysis comparing the percentage of HNF4α+ cells in contact (vimentin+) or remote from sites of contact with hHSCs (vimentin−) (*n* = 4). Total of 6.69 × 10^5^ cells analyzed. (D–F) Functional bioassay for FGF7 signaling reveals a requirement for direct coculturing of hHSCs with target cells. (D) RT-qPCR showing the time course response of HepaRG cells (day 13) to a single dose of vehicle (cont) or rhFGF7. Changes in *APOA2* expression at 48 h were used as a biological readout for FGF7 activity in E–F. (E) Conditioned medium from MitoC-treated hHSCs was insufficient to illicit an *APOA2* response at 48 h, whereas (F) direct addition of hHSCs to HepaRG cultures activated *APOA2* expression in an FGF7-dependent manner, as judged by the ability of rhFGFR2-IIIb-Fc to block induction. Importantly, this effect was conditional upon prior treatment of hHSCs with MitoC, thus confirming the positive effect of mitotic inhibition on the ability of hHSCs to communicate with LPCs via FGF7. Data information: underlying data are available in S1 Data. Data are presented as mean + SEM. *P < 0.05, **P < 0.01, and ***P < 0.001. (A) Paired Student *t* test. (B, E, F) One-way ANOVA was used to compare means. Significance *P* values were calculated using Tukey's multiple comparison test. (D) Two-way ANOVA was used to compare means. Significance *P* values were calculated using Tukey's multiple comparison test. *APOA2*, apolipoprotein A2; cont, control; Fgfr2-IIIb, Fgf7 receptor; Fgf7, fibroblast growth factor 7; hHSC, human HSC; HNF4α, hepatocyte nuclear factor 4-alpha; HSC, hepatic stellate cell; *Irs2*, insulin receptor substrate 2; LPC, liver progenitor cell; MitoC, mitomycin C; mRNA, messenger RNA; rhFGFR2-IIIb-Fc or rhIIIb-Fc, recombinant protein comprising human Fgfr2-IIIb extracellular domain fused to Human IgG1 Fc domain used to inhibit FGF7 binding its receptor; rhFGF7, recombinant human Fgf7; RT-qPCR, reverse transcriptase-quantitative PCR; RU, relative unit.

In order to further test whether paracrine FGF7 signaling was downstream of *IRS2*-dependent stromal–epithelial crosstalk in hHSC/HepaRG cocultures, we used primary hHSCs, which expressed higher levels of endogenous FGF7 than LX-2 (Fig 8). Interestingly, we found that both *FGF7* and *IRS2* expression were significantly increased in hHSCs following MitoC-induced cell-cycle arrest (Fig 8A). This observation provided us with a useful means to modulate endogenous FGF7 expression prior to coculturing the cells with HepaRG. Consistent with the increase in *FGF7* expression, we observed a dramatic enhancement of *APOA2* expression in HepaRG cocultures using hHSCs pretreated with MitoC (Fig 8B). Moreover, inclusion in the medium of a competitive FGF7 inhibitor, consisting of a chimeric rFGFR2-IIIb extracellular domain/Human IgG1 crystallizable fragment (Fc) domain protein (rhFGFR2-IIIb-Fc), significantly inhibited *APOA2* expression by HepaRG, confirming APOA2 as a downstream target of paracrine FGF7 and demonstrating a role for FGF7 signaling in driving hepatocyte differentiation in coculture.

The diffusion of FGF7 is highly restricted because of its affinity for heparin-sulphate proteoglycans in extracellular matrix and cell membranes [23]. Consistent with this short-range mode of action, we found that hepatocyte differentiation within the cocultures (judged by HNF4α immunostaining) was spatially limited to sites of contact between HepaRG and vimentin+ hHSCs (Fig 8C). To explore the properties of the HSC signal driving differentiation within the cocultures further, we developed a short-term bioassay using *APOA2* expression as a functional readout for FGF7 activity. Stimulation of HepaRG monocultures with a single dose of rhFGF7 rapidly induced *APOA2* at 48 h (Fig 8D). Hence, we collected conditioned medium from MitoC-treated hHSCs and added it to HepaRG cells; however, no induction of *APOA2* was observed, suggesting the factor was either labile or unavailable in the cellular supernatant because of cell/matrix binding (Fig 8E). Using a similar approach, we were able to confirm that direct contact between HepaRG and hHSCs (achieved by short-term 48 h coculture) was sufficient to induce *APOA2* expression in an FGF7-dependent manner, an effect that was blocked by inclusion of the competitive FGF7 inhibitor (Fig 8F). Importantly, the ability of hHSCs to induce *APOA2* within 48 h in this model was conditional upon the pretreatment of hHSCs with MitoC, confirming that paracrine FGF7 signaling in hHSCs was enhanced by mitotic inactivation.

Taken together, these data demonstrated a positive role for paracrine FGF7 signaling by human HSC in supporting FGF7 sensitivity and hepatocyte differentiation in adjacent epithelial progenitors.

### *IRS2* deficiency promotes stromal activation at the expense of epithelial repair

The stromal injury response precedes epithelial repair. In the liver, this is characterized by HSC-derived myofibroblast proliferation and the laying down of extracellular matrix, which is followed by cell-cycle exit, fibrogenic reversion, and apoptotic cell clearance that accompanies tissue repair. The equilibrium within the fibrotic stroma between activated and reverted HSCs is therefore central to the regulation of the stromal injury response and has important consequences for liver fibrosis and scarring in the context of chronic injury.

**Fig 9:**
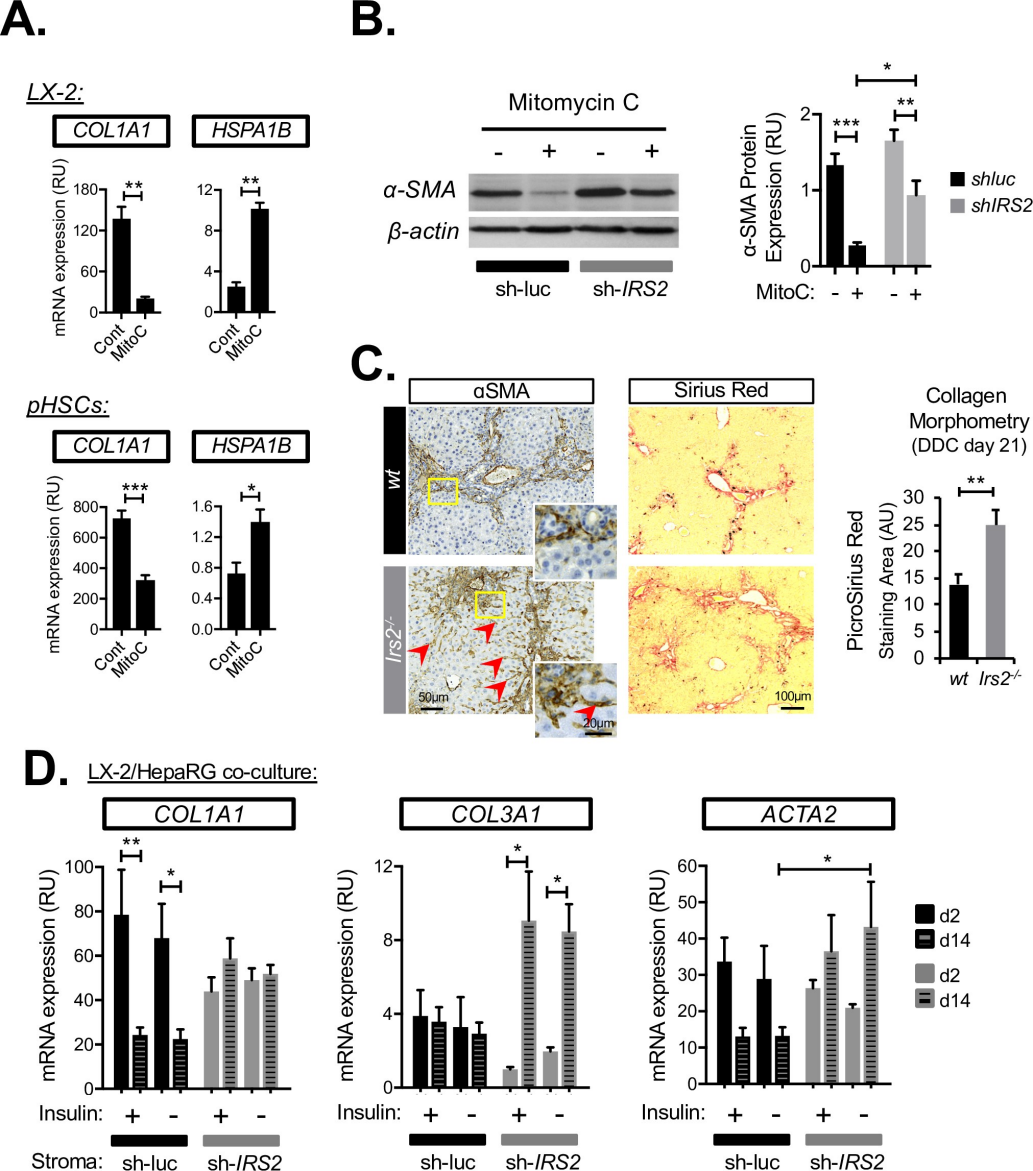
Loss of IRS2 promotes stromal activation and inhibits HSC reversion. (A–B) Cell-cycle arrest induced by MitoC leads to fibrogenic reversion of hHSCs, which was delayed by *IRS2* silencing. (A) RT-qPCR analysis of LX-2 cells and primary hHSC cultures treated with vehicle (cont) or MitoC for 72 h prior to harvesting showing decreased *COL1A1* expression together with concomitant increase in HSC reversion marker HSPA1B (*n* = 3). (B) Western blot analysis of αSMA protein down-regulation in cont (sh-luc) and silenced (sh-*IRS2*) LX-2 cells during MitoC treatment (72 h) (*n* = 4–5). (C) Hepatic fibrosis after 21 days of DDC treatment was exacerbated in *Irs2*^−/−^ mice. (Left) Representative immunohistochemical images of control (WT) and *Irs2*^−/−^ livers for myofibroblast marker αSMA together with Picro Sirius Red histochemistry used for collagen morphometry; quantified in graph (right) (*n* = 8). Red arrowheads highlight pattern of increased perisinusoidal fibrosis that was more pronounced in *Irs2*^−/−^ livers. (D) RT-qPCR analysis of cont (sh-luc) and IRS2-deficient (sh-IRS2) LX-2 cocultures with HepaRG cells performed in the presence (+) or absence (−) of supplemented insulin. Fibrogenic gene expression was analyzed at 2 days and 14 days after seeding the two cell types together (*n* = 3). Data information: underlying data are available in S1 Data. Data are presented as mean + SEM. *P < 0.05, **P < 0.01, and ***P < 0.001. (A) Paired Student *t* test. (B and D) Two-way ANOVA was used to compare means. Significance *P* values were calculated using Sidak's multiple comparison test. (C) Unpaired Student *t* test. *Acta2*, gene encoding αSMA; AU, arbitrary unit; *COL1A1*, Collagen 1A1; cont, control; hHSC, human HSC; HSC, hepatic stellate cell; HSPA1B, heat shock 70 kDa protein 1B; *Irs2*, insulin receptor substrate 2; MitoC, mitomycin C; mRNA, messenger RNA; pHSC, primary human HSC; RT-qPCR, reverse transcriptase-quantitative PCR; RU, relative unit; shIRS2, shRNA-targeting IRS2; sh-luc, control luciferase; shRNA, short hairpin RNA; WT, wild type; αSMA, alpha-smooth muscle actin.

Our data showed that both *FGF7* and *IRS2* expression were induced in hHSCs upon triggering of cell-cycle arrest using MitoC (Fig 8A). MitoC treatment was also accompanied by a phenotypic shift in hHSCs and LX-2s consistent with fibrogenic reversion (Fig 9A), as judged by loss of Collagen 1A1 (*COL1A1*) expression and activation of antiapoptotic heat shock 70 kDa protein 1B (*HSPA1B*), previously identified as a biomarker of HSC reversion [31]. Importantly, the down-regulation of myofibroblast marker protein αSMA upon cell-cycle exit was delayed in LX-2 cells in which *IRS2* was silenced (Fig 9B), suggesting impaired transition from an activated to “reverted” state. These in vitro data, which suggested that *IRS2* could participate in the transition of HSCs to a “reverted” phenotype, were consistent with the in vivo observation that DDC-treated *Irs2*^−/−^ mice had increased periportal and perisinusoidal fibrosis, judged by Sirius Red collagen morphometry and αSMA immunostaining (Fig 9C).

**Fig 10:**
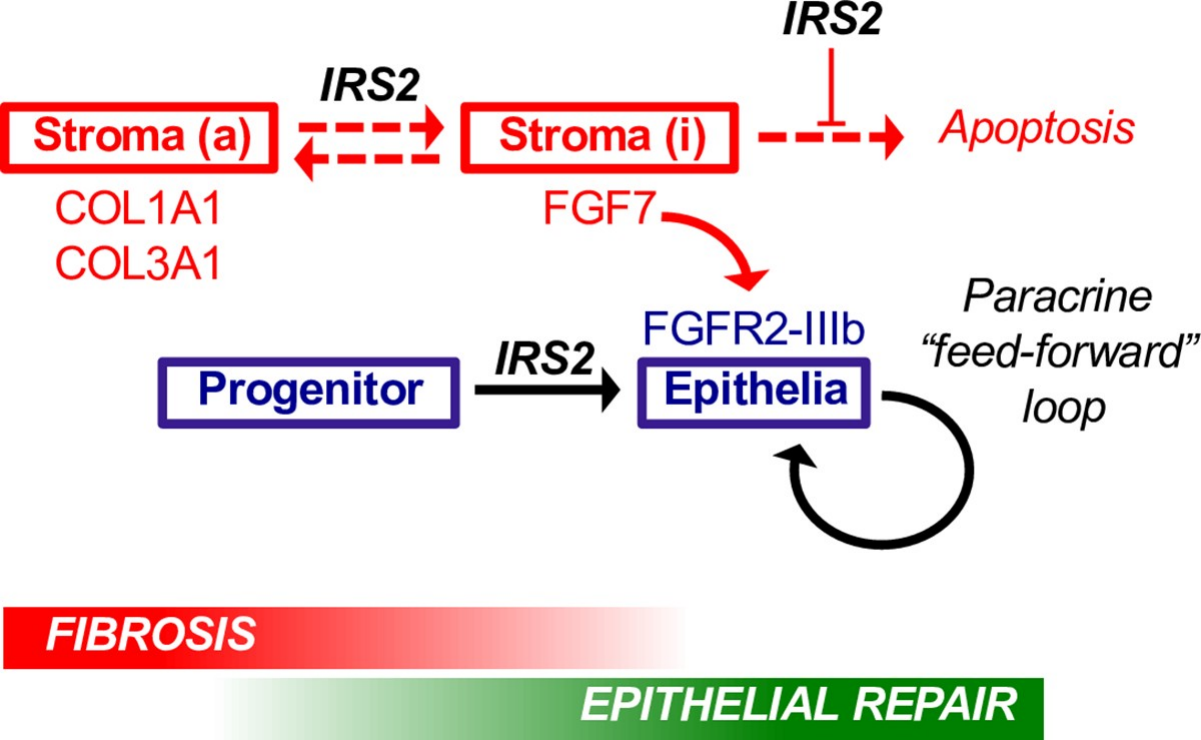
IRS2 promotes epithelial repair in the liver by potentiating HSC–LPC crosstalk. Model summarizing the major findings of our study in stromal (red) and epithelial (blue) cell compartments. IRS2 promoted reversion of activated (a) fibrogenic stroma and survival of inactivated (i) cells expressing high levels of FGF7. In progenitors, IRS2 was required for differentiation and *FGFR2-IIIb* expression—priming sensitivity to FGF7. Epithelial sensitivity to FGF7 was then further increased by the ability of the FGF7 ligand to increase FGFR2-IIIb expression, resulting in a feed-forward paracrine loop capable of sustaining epithelial repair by potentiating heterotypic cell–cell communication. Fgfr2-IIIb, Fgf7 receptor; Fgf7, fibroblast growth factor 7; *Irs2*, insulin receptor substrate 2; HSC, hepatic stellate cell; LPC, liver progenitor cell.

We therefore returned to the LX-2/HepaRG cocultures to assess changes in fibrogenic markers. Using this method, we found that upon coculture with LPC-like cells, control (sh-luc) LX-2 cells underwent a process of time-dependent fibrogenic reversion, during which fibrillar collagen (*COL1A1)* expression was significantly down-regulated and αSMA/*ACTA2* dampened (Fig 9D). In striking contrast to this, we found that silencing of *IRS2* in LX-2 cells abrogated their ability to suppress *COL1A1* in coculture, and we instead observed a dramatic induction of nonfibrillar type III collagen (*COL3A1*) indicative of an activated stromal injury response. Time-dependent switching from myofibroblast marker THY1 to LPC-associated SPP1 was also dampened by silencing of *IRS2* in the stroma (S10B Fig), and the numbers of GFP-expressing LX-2 cells at the end of the cocultures was increased (S10C Fig). Interestingly, unlike LPC and epithelial repair genes (*SPP1*/*APOA2*/*FGFR2-IIIb*), whose expression in coculture was both insulin and IRS2 dependent (Fig 7C and S10 Fig), changes in fibrogenic/myofibroblast genes *COL1A1*, *COL3A1*, *ACTA2*, and *THY1* (Fig 9D and S10 Fig) were largely unaffected by omission of insulin from the medium, suggesting the ability of IRS2 to restrain activation of the fibrogenic stroma was insulin independent. We concluded that silencing of *IRS2* in HSCs prolonged their fibrogenic activation in coculture while slowing fibrogenic reversion by MitoC and reducing the survival of mitotically inactivated cells in which FGF7 expression was increased. Thus, IRS2 signaling in the fibrotic stroma served as a switch between states of stromal activation and fibrogenic reversion that potentiated heterotypic paracrine signaling between the two cell types and drove epithelial FGF7 sensitivity in LPCs. Together with our results demonstrating a cell-intrinsic role for *IRS2* in LPCs in promoting FGF7 sensitivity, we proposed a model in which IRS2 exerted multiple proregenerative effects in both stromal and LPC cell compartments that favored epithelial repair over fibrogenesis during chronic liver injury (Fig 10).

## Discussion

In this study, we provide compelling evidence that *Irs2* is required for hepatic wound healing and epithelial repair during chronic DDC feeding in mice and for sustaining local Fgf7/Fgfr2-IIIb signaling. Using human cell models, we have shown that *IRS2* can affect multiple processes in both the fibrotic stroma and bipotent LPC-like cells that impinge upon their abilities to communicate with one another via FGF7 signaling. In addition, coculture studies confirm that *IRS2* deficiency in HSCs disrupts paracrine crosstalk between the stromal niche and LPCs, resulting in failure to induce *FGFR2-IIIb* and epithelialization while simultaneously prolonging fibrogenic activation. Our data therefore support the hypothesis that insulin resistance could lead to defects in hepatic injury repair that exacerbate liver damage and progression of chronic liver disease because of reduced paracrine FGF signaling. These data also have profound implications for our understanding of how systemic insulin signals are interpreted by local injury-repair mechanisms and demonstrate that defects in insulin sensitivity can influence short-range communication between distinct cell types within a tissue, notably between the so-called “niche” and adult stem cells responsible for producing new epithelia.

Interestingly, insulin resistance in human liver disease is associated with increased fibrosis and the appearance of reactive periportal lesions called "ductular reactions" (DRs), in which intermediate “hepatobiliary” progenitors expand surrounded by fibrotic stroma [32]. These lesions exhibit striking similarities with those observed in the DDC-injury model [33], although DRs are a feature of disrepair because of their correlation with poor prognosis. The association between insulin resistance and DRs has been demonstrated in liver disorders including NASH and chronic hepatitis C [32,34], in which defective *IRS2* expression has also been reported [10,11]. Our results suggest that reduced sensitivity to insulin in patients with chronic liver disease could alter the intrinsic abilities of LPCs to contribute to regenerative processes and lead to a derangement of heterotypic cell–cell interactions that mediate their expansion and differentiation, leading to DRs. Our discovery that *IRS2* silencing in HSCs also promoted fibrogenesis while limiting hepatocyte differentiation and FGFR2-IIIb expression in coculture recapitulated the increase in liver fibrosis and reduced Fgf7/Fgfr2-IIIb expression described in *Irs2*^−/−^ mice during DDC treatment. Hence, our data suggest that lRS2 signaling may serve as a switch that regulates the transition between phases of stromal and epithelial expansion within DRs by promoting reversion and survival of FGF7-expressing stroma while simultaneously priming LPCs to express FGFR2-IIIb (Fig 10).

There is precedent that IRS proteins play an important role in driving epithelial growth via modification of the stromal microenvironment in the liver. NT-157, the allosteric inhibitor of IRS1/2, was recently found to limit colorectal cancer metastasis to the liver by reducing the trophic support received by stromal myofibroblasts or HSCs [35]. These findings exhibit striking parallels with our results and highlight the potential future impact of our feed-forward paracrine model in the field of cancer biology, in which subversion of injury-repair mechanisms by tumors are well documented. Consistent with this, Irs2 was also recently implicated in a positive feedback loop driving disease progression and hepatocarcinogenesis in rodent models of nonalcoholic fatty liver disease (NAFLD) [13]. Interestingly, the study identified hepatic *Irs2* expression as a downstream target of Yes-associated protein (YAP) and Tafazzin (TAZ)—two oncogenic effectors of the Hippo signaling pathway that reversibly control hepatocellular fate [36], LPC activation [37], and liver regeneration [38]. Hence, it seems likely that subversion of Irs2-dependent aspects of the LPC response identified in our study also contribute to the pathophysiology of metabolic liver disease and cancer.

Insulin and FGF7 synergized via *IRS2* to promote osteopontin/*SPP1* expression and sustain hepatocyte differentiation, thus revealing a cell-intrinsic role for insulin/IRS2 in priming and potentiating LPC sensitivity to paracrine FGF7 and other IIIb ligands such as FGF22 and FGF10. Insulin/*IRS2* signaling was required for the formation of “islands” in HepaRG cultures, which expanded because of a process of hepatocyte differentiation and epithelialization and conferred FGF7 sensitivity to the cultures by promoting FGFR2-IIIb expression. Conversely, in DDC-treated *Irs2*^−/−^ mice, the numbers of hepatocytes declined, and the process of re-epithelialization was impaired, as manifested by delayed restoration of the parenchymal architecture, delayed induction of *Epcam*, failure to sustain *Fgfr2-IIIb*, and failure to expand numbers of small hepatocytes. Thus, it is tempting to speculate that *IRS2* promotes hepatocyte differentiation of LPCs in vivo, although this would need to be empirically tested.

We have identified positive feedback between FGF7 and its receptor in HepaRG monocultures as well as HepaRG/HSC cocultures and demonstrated a role for *IRS2* in both cell compartments upstream of FGFR2-IIIb amplification (Fig 10). These data were highly consistent with the observed dual failure of Fgf7/Fgfr2-IIIb in *Irs2*^−/−^ mice during injury and provide new insight into the underlying synergies that help promote epithelial sensitivity to proregenerative stromal growth factors such as Fgf7. Positive feedback between Fgf10 and *Fgfr2-IIIb* has previously been reported during salivary gland development [15], and in nonmalignant tumors, Fgf7-mediated FGFR2-IIIb expression is thought to play a tumor-suppressive role by promoting epithelial differentiation [23,39]. Interestingly, synergy between Fgfr2-IIIb ligands and their receptor has previously been linked to crosstalk with phosphoinositide 3-kinase (PI3K)—one of the principle effectors of insulin/IGF-1 signaling recruited by IRS2-binding [15]. Indeed, inhibition of PI3K/Protein kinase B (AKT) limits hepatocellular reprogramming by Fgf10/Fgfr2-IIIb during DDC injury in vivo [22], suggesting Irs2 could act upstream of this pathway in promoting cellular plasticity in the liver.

Further to impairment of Fgf7, we also describe reductions in *Fgf10* during DDC injury and in LX-2/HepaRG cell coculture. Hence, we cannot rule out the possibility that the IRS2-dependent paracrine feed-forward loop we have identified also involves other stromal FGFR2-IIIb ligands. HSC-derived Fgf10 plays an important role in the expansion of bipotent LPCs during liver development [40], and both FGF10 and 22 are induced by the fibrotic stroma during DDC injury [22]. A deeper understanding of the indirect nature of how IRS2 regulates these genes within the stroma is therefore needed.

In recent years, FGFs have received increasing attention for their unexpected role in metabolic disease and for their potential to treat metabolic disorders by helping restore insulin sensitivity. FGF1, 19, and 21 have potent (albeit poorly understood) effects on metabolism and insulin signaling, largely through their putative actions in white adipose tissue and the liver [21,41], whereas impaired FGF-receptor signaling is associated with insulin resistance, liver disease [41,42], and impaired hepatic regeneration [43,44]. Blunted expression of FGF7 has also been previously linked to delayed skin wound healing in genetically diabetic leptin deficient (db/db) mice [45,46]. Our data therefore raise the intriguing possibility that a comparable wound-healing defect may also exist in the livers of *Irs2*^−/−^ mice following injury, resulting in a breakdown in stromal–epithelial paracrine signaling and consequent failure to coordinate the injury response. Combined future strategies to potentiate hepatic insulin sensitivity and FGF7 signaling may therefore improve patient outcomes by promoting epithelial repair in the liver.

The impact of insulin resistance on the stromal microenvironment in the liver during injury is poorly understood [5], as is the role played by IRS proteins in regulating HSC biology, which involves injury-dependent cycles of myofibroblast activation and cell clearance during so-called "fibrogenic reversion." The model we present to explain impaired stromal induction of Fgf7 in the *Irs2*^−/−^ mice (Fig 10) incorporates how IRS2 influenced fibrogenic reversion and survival of HSCs—only one of several FGF7-expressing stromal subpopulations that respond to DDC injury. However, we also provide evidence that the cellular composition of the fibrotic milieu was different in the livers of *Irs2*^−/−^ mice and leave open the possibility that *Irs2*-deletion affected other resident FGF7-expressing cells such as PFs. Finally, we observed a greater increase in T cells and the incorporation of bloodborne mesenchymal cells, possibly to the detriment of resident stromal cells or simply to compensate for the failure of FGF7-expressing stroma. Fibrocytes from type II diabetic patients have recently been shown to display altered migratory and inflammatory characteristics that could also have contributed to the altered pattern of stromal gene expression and impaired wound healing in our model [47].

We show that DDC injury had a negative impact on Gfap+ HSCs in *Irs2*^−/−^ mice. Gfap is associated with quiescent or inactivated HSCs; hence, our data are consistent with accelerated turnover/loss of these cells in the livers of *Irs2*^−/−^ mice due to a combination of reduced survival and/or reduced myofibroblast reversion (Fig 10). Our investigation using hHSCs revealed that *IRS2* protected cells from apoptosis following mitotic inactivation using MitoC, providing a mechanism to explain the dampening of Fgf7 and increased apoptosis observed in the livers of *Irs2*^−/−^ mice during injury. Interestingly, both *IRS2* and *FGF7* were increased in primary hHSCs upon cell-cycle arrest and fibrogenic reversion while enhancing their ability to drive epithelial differentiation of HepaRG cells in an FGF7-dependent manner in coculture. Similarly, IRS2 was required in LX-2 cells to sustain both FGF7 expression and to allow a process of fibrogenic reversion to proceed in the presence of HepaRG cells. These data provide new evidence to suggest that epithelial repair signals produced by HSCs are increased by a program of cell-cycle arrest or cell death, two processes that are more closely aligned with stromal reversion than myofibroblast activation. Given that the regulation of *FGF7* expression by the fibrotic stroma is poorly understood, we propose the hypothesis that fibrogenic reversion, rather than myofibroblast activation, drives FGF7 expression during injury and that IRS2 preferentially favors reversion and survival of HSCs tasked with stimulating epithelial repair. Future work by this laboratory will seek to further test this hypothesis.

## Materials and methods

### Ethics statement

Mice were housed in the facility of the CIPF (Valencia, Spain), which is registered as an experimental animal Breeding, User, and Supplier Center (reg. no. ES 46 250 0001 002) under current applicable Spanish regulations (RD 53/2013). Animals were kept in ventilated racks with microisolators under specific-pathogen–free (SPF) conditions and handled by accredited personnel and treated in accordance with EU legislation and welfare guidelines. Procedures were approved by the CIPF "Comite Ético de Experimentación Animal" (CEEA) and the ministry for "Agricultura, pesca, alimentacion y agua" of the Generalitat Valenciana (Valencia, Spain), under the authorization code 2014/VSC/PEA/00062 tipo 2. Mice were killed by fentanil/pentobarbital overdose followed by cervical dislocation.

### Animal experimentation

WT and Irs2^−/−^ C57BL/6 littermates (females aged 10–12 weeks, fasting blood glucose < 100 mg/dL) were fed a modified LabDiet (5015) containing 0.1% DDC (TestDiet, St. Louis, MO, USA). Animals were killed at intervals up to 21 days during the light cycle. FGF7 stimulation was performed by i.p. injection of (0.3 mg/kg) recombinant mouse KGF (Taper BioLegend, San Diego, CA, USA).

### Liver injury and cholestasis markers

Serum transaminases were measured by ELISA according to manufacturer’s instructions (MilliporeSigma, St. Louis, MO, USA), and serum total bilirubin (TBIL) was measured by colorimetric assay according to the Jendrassik-Grof method (MilliporeSigma). Bile acids were measured by metabolomic analysis. Briefly, samples were extracted from whole-liver tissue using the metanol-chloroform-H_2_O method [48] and analyzed on a 600 MHz NMR spectrometer equipped with a cryoprobe. Metabolites were identified with the aid of the spectral databases HMBD [49] and BMRB [50] and quantified using MestreNova8.

### Cell culture

HepaRG cells (kind gift of Anne Corlu and Cristiane Guillouzo) were passaged and differentiated as previously described [30] using Williams E medium (10% fetal calf serum, 50 μM hydrocortisone hemisuccinate, 0.88 μM insulin, and 2 mM L-glutamine and penicillin-streptomycin). DMSO (2%) was added from day 14. Insulin supplement was omitted from the medium where indicated 24 h postplating. rhFGF7 (50 ng/ml; Cell Guidance Systems, St. Louis, MO, USA) or rhFGFR2-IIIb Fc Chimera (100 ng/ml; R&D Systems, Minneapolis, MN, USA) were added to media where indicated every 48 h. Cryopreserved primary hHSCs were obtained from a healthy 15-year-old Caucasian female donor using a previously described isolation method [51] (Innoprot, Derio, Spain). LX-2 cells (kind gift of Scott Friedman) and primary hHSCs were cultured in DMEM (2% fetal calf serum), and primary HSCs were used within 5 passages. LX-2/HSCs were inactivated for 3 h with 1mg/ml MitoC (MilliporeSigma) before seeding onto 0.1%-gelatin–coated plates. After 24 h, activated/inactivated HSC cultures were switched to HepaRG medium until the end of the experiments. Stable cell lines were generated by lentiviral transduction (vectors summarized in S1 Table) using a multiplicity of infection of 0.5–20 TU/cell in the presence of 8 μM polybrene. Infections were performed 16 h postplating for 6 h in serum-free medium. Where applicable, cells were selected for 7 days in media containing 2.5 μg/ml puromycin.

### Cell viability

MTT assay: 3-(4,5-dimethyl-2-thiazolyl)-2,5-diphenyl-2-H-tetrazolium bromide (5 mg/ml, MilliporeSigma) was added to media in a 1:5 ratio and incubated in the dark for 4 h. Resulting formazan crystals were resuspended in DMSO, and optical density (OD) was measured at 570 nm using an automated microplate reader (Victor; PerkinElmer, Waltham, MA, USA).

### Fluorescence immunostaining

Fresh frozen liver tissue sections (6 μm, mounted in OCT, MilliporeSigma) or cells were fixed using 4% paraformaldehyde–PBS, washed, and permeabilized with 0.5% Triton-X100 before blocking (1% BSA, 5% horse serum, 0.2% Triton-X100). Primary antibodies (S2 Table) were incubated over night at 4 °C. Alexa-conjugated secondary antibodies (Invitrogen, Carlsbad, CA, USA) were applied for 1 h together with 5 μg/ml Hoechst 33342 prior to mounting. Confocal images were obtained using a Leica TCS-SP6 (Leica, Wetzlar, Germany). Fluorescence image analysis was performed using INCell Analyzer 1000 (GE Healthcare, Chicago, IL, USA) as outlined below.

### Immunohistochemistry

Formalin-fixed paraffin-embedded tissue sections (4 μm) were processed automatically using the Autostainer Link 48 (Agilent, Santa Clara, CA, USA) with EnVision FLEX reagents according to manufacturers’ instructions (Agilent). Antigen retrieval was performed using high-pH target retrieval solution (Agilent). Antibodies are provided in S2 Table.

### Collagen morphometry

Collagen was visualized in PFE liver sections using the Picro Sirius Red technique (Abcam, Cambridge, UK) according to manufacturer’s instructions. Following imaging, threshold analysis was performed using FIJI software to quantify staining in a total area comprising >10 portal fields per animal.

### Image analysis

Fluorescence immunostained cells/cryosections were analyzed using the “INcell 1000” imaging platform and software (GE Healthcare) where indicated. Individual cells were identified by Hoechst staining. Threshold analysis was used to determine the expression of the following markers: *pAPOA2*-GFP, albumin, CYP3A4, HNF4α, Ki67, Spp1, and Vimentin. For replicate well/tissue sections, at least 20 fields were randomly analyzed for each replicate. PFE tissue sections were imaged using Pannoramic Viewer (3DHISTEC) and analyzed with FIJI software; β-catenin staining was quantified using FIJI (histogram function) to calculate modal pixel intensity.

### Hepatocyte ploidy

Interpolation of DNA content in HNF4α/Hoechst-stained liver sections was performed using a methodology developed by our laboratory using INcell 1000 (GF Healthcare) that enabled in situ approximation of hepatocyte ploidy (S3 Fig). DNA content was calculated for all circular hepatocyte nuclei (nuclear elongation value > 0.8) as a combined function of Hoechst nuclear fluorescence intensity (DNA density) and nuclear volume (calculated as a spherical function of nuclear radius). Hepatocyte ploidy was then calibrated using circular HNF4α− NPC cell population within the tissue as a 2n control. The 2c hepatocyte population was defined as the population of HNF4α+ nuclei within the 19.99 μm^2^–34.99 μm^2^ nuclear size range with a nuclear circularity index >0.8.

### Immunoblotting

Tissues were lysed in RIPA buffer containing complete protease and phosphatase inhibitors (Roche, Basel, Switzerland). Protein (20 μg/well) was separated using standard SDS-PAGE. Transferred PVDF membranes were blocked (TBS–Tween 3% BSA) and probed with primary antibodies (S2 Table).

### RT-qPCR

Total RNA was extracted using RNeasy Mini Kit (Qiagen, Venlo, The Netherlands) with DNaseI Digestion. Liver tissue was preprocessed using Trizol (MilliporeSigma). For experiments using cells, RNA from duplicate wells were analyzed. First-strand synthesis was performed using EcoDry Premix (Takara, Kusatsu, Japan), and real time-PCR was carried out in LightCycler 480 II (Roche) using SYBR PreMix ExTaq (Mi RNaseH Plus, Takara). Reverse transcriptase-quantitative PCR (RT-qPCR) was performed in triplicates for all RNAs analyzed. Primers are listed in S3 Table. Relative gene expression was calculated by normalization to Glyceraldehyde-3-Phosphate Dehydrogenase (GAPDH) in mouse liver and to Ribosomal Protein L19 (RPL19) in human cell lines.

### Statistics and data processing

For experiments using animals, values of *n* reflect the number of animals per cohort. Cohort size was based on previous studies and expertise using the Irs2^−/−^ model. For nonanimal experiments, values of *n* reflect the number of independent experiments performed. When possible, automated methods of image analysis were employed to quantify immunostainings using the “INcell 1000” imaging platform and software (GE Healthcare). Statistical analyses were performed with Prism version 7.00 for Windows, GraphPad Software, La Jolla, CA, USA, www.graphpad.com. One-way ANOVA was used to compare multiple means with one variable. Multiple comparisons were obtained applying Tukey’s test. Ordinary two-way ANOVA was used to analyze data sets with several variables and Bonferroni’s test for multiple comparisons. When two data sets were compared, unpaired Student *t* test was used. Results were represented as mean + SEM considering statistical significance: *P < 0.05, **P < 0.01, and ***P < 0.001.

## Supporting information

S1 Fig Impact of DDC injury on Fgfr2-IIIb ligand gene expression. Whole-liver mRNA levels were assessed for Fgf7 family member genes *Fgf10* and *Fgf22* in WT and *Irs2*^−/−^ mice during a time course of DDC feeding by RT-qPCR (*n* = 3–8). Data information: underlying data are available in S2 Data. Data are represented as mean + SEM: Two-way ANOVA was used to compare means. Significance *P* values were calculated using Tukey’s multiple comparison test (*P < 0.05). Dotted lines indicate statistically significant decrease with time. DDC, 3.5-diethoxycarbonyl-1.4-dihydrocollidine; Fgfr2-IIIB, Fgf7 receptor; Fgf7, fibroblast growth factor 7; *Irs2*, insulin receptor substrate 2; mRNA, messenger RNA; RT-qPCR, reverse transcriptase-quantitative PCR; WT, wild type.
(TIF)

S2 Fig Delayed expansion of NPCs parallels exacerbated parenchymal cell depletion in the livers of *Irs2*^−/−^ mice during DDC feeding. (A–C) HNF4α immunostaining was used to quantify the parenchymal (HNF4α+) and NPC (HNF4α−) responses to DDC liver injury in WT and *Irs2*^−/−^ mice. (A) Representative images of HNF4α immunostaining (DDC day 21). Below: masks used to gate and quantify hepatocyte and NPC densities in whole-liver sections using INcell Analyzer (total cells analyzed: 1.23 × 10^6^). (B) Time course of DDC liver injury comparing the numbers of NPC nuclei (HNF4α−) in livers of WT and *Irs2*^−/−^ mice (*n* = 4–6, total of 7.2 × 10^5^ HNF4α− nuclei analyzed). (C) Time course of DDC liver injury comparing the numbers of hepatocyte nuclei (HNF4α+) in livers of WT and *Irs2*^−/−^ mice (*n* = 4–6, total of 5.1 × 10^5^ HNF4α+ nuclei analyzed). (D) Size distribution of HNF4α+ hepatocyte nuclei in livers of WT and *Irs2*^−/−^ mice following DDC liver injury (d21), calculated in situ using INCell Analyzer. Data show significant depletion of small hepatocytes nuclear area < 75 μm^2^. (*n* = 4, total of 1.2 × 10^5^ HNF4α+ nuclei analyzed). Data information: underlying data are available in S2 Data. Data are presented as mean + SEM. *P < 0.05, **P < 0.01, and ***P < 0.001. Two-way ANOVA was used to compare means. Significance *P* values were calculated using Bonferroni test. DDC, 3.5-diethoxycarbonyl-1.4-dihydrocollidine; HNF4α, hepatocyte nuclear factor 4-alpha; *Irs2*, insulin receptor substrate 2; NPC, nonparenchymal cell; WT, wild type.
(TIF)

S3 Fig Loss of hepatocytes in *Irs2*^−/−^ mice during chronic DDC liver injury corresponds with failure to activate small “2c” hepatocyte populations. (A–B) Analysis of hepatocyte ploidy in HNF4α immunolabelled liver sections. (A) High-content imaging was used to generate frequency, size, and morphometry profiles of HNF4α+ nuclei based on quantification of Hoechst DNA staining in liver tissue sections. Upper panel shows representative images of hepatocyte nuclei of different sizes and shapes, with smaller nuclei (<75 μm^2^) tending to have greater circularity indices (>0.8), whereas larger nuclei tended to be bilobular. We observed discrete peaks in nuclear “circularity” (I–IV) that we hypothesized corresponded to the major ploidy groupings in the liver: 2n, 4n, 8n, and 16n. Data shown from untreated WT livers (*n* = 4, 1.35 × 10^4^ HNF4α+ nuclei per animal). (B) To test this hypothesis, all nuclei were gated for circularity (>0.8), and DNA content was calculated for peaks I–V as a function of interpolated nuclear volume and Hoechst intensity (formula below). Using HNF4α− NPCs as an internal 2n control, we confirmed that populations I–IV accurately represented 2c, 4c, 8c, and 16c hepatocyte populations, respectively (*n* = 4, 1.1 × 10^4^ HNF4α+ nuclei per animal). This original methodology to describe hepatocyte ploidy in situ was then applied to WT and Irs2^−/−^ livers during DDC feeding. (C) Quantification of small hepatocytes with an estimated 2n DNA content (2c) as calculated in situ using INCell Analyzer showing time-dependent increase in WT livers (days 14–21) and significant depletion in livers of *Irs2*^−/−^ mice following DDC feeding (day 21) (*n* = 4–6, total of 4.8 × 10^4^ HNF4α+ nuclei analyzed). Data information: underlying data are available in S2 Data. Data are presented as mean + SEM. *P < 0.05, **P < 0.01, and ***P < 0.001. Two-way ANOVA was used to compare means. Significance *P* values were calculated using Bonferroni test. DDC, 3.5-diethoxycarbonyl-1.4-dihydrocollidine; HNF4α, hepatocyte nuclear factor 4-alpha; *Irs2*, insulin receptor substrate 2; NPC, nonparenchymal cell; WT, wild type.
(TIF)

S4 Fig PF/myofibroblast markers are expressed at equivalent or increased levels in livers *Irs2*^−/−^ mice after DDC injury. (A–B) Analysis of PF/myofibroblast markers by immunostaining in periportal sections of WT and *Irs2*^−/−^ mice on day 21 of DDC feeding. (A) Representative confocal images of DDC livers using indicated antibodies. Dotted line = portal vein. (B) Graphical quantification of immunostainings by analysis of staining area (*n* = 3–4). Data information: underlying data are available in S2 Data. Data are presented as mean + SEM. *P < 0.05. (B) Unpaired Student *t* test was used to compare means. DDC, 3.5-diethoxycarbonyl-1.4-dihydrocollidine; *Irs2*, insulin receptor substrate 2; PF, portal fibroblast; WT, wild type.
(TIF)

S5 Fig The stromal niche in *Irs2*^−/−^ mice is replete with cells expressing PF/myofibroblast markers Thy1 and αSMA but exhibits reduced contact between Gfap^+^ HSCs and LPCs. (A–B) Confocal images of immunofluorescence-stained DDC livers describing the stromal environment surrounding LPCs in WT and *Irs2*^−/−^ mice at the indicated time points. White dotted line = portal vein. (A) Thy1+ cells surrounded ducts containing Epcam+/Spp1+ LPCs in the livers of both WT and *Irs2*^−/−^ mice (indicated by arrows). Selected images are representative of *n* = 5. (B) The stromal niche in both WT and *Irs2*^−/−^ mice also contained αSma+ myofibroblasts (arrowheads) surrounding Spp1+ LPCs. However, Gfap+ HSCs were reduced in number in the *Irs2*^−/−^ stroma, and contact between Gfap+ cells and LPCs (arrows) was reduced. Yellow dotted boxes mark expanded regions of interest containing representative duct-like structures (*). Selected images are representative of *n* = 3–5. DDC, 3.5-diethoxycarbonyl-1.4-dihydrocollidine; EpCAM, epithelial cell adhesion molecule; Gfap, glial fibrillary acidic protein; HSC, hepatic stellate cell; *Irs2*, insulin receptor substrate 2; LPC, liver progenitor cell; PF, portal fibroblast; Spp1, secreted phosphoprotein 1; Thy1, Thy-1 cell surface antigen; WT, wild type; αSma, alpha-smooth actin muscle.
(TIF)

S6 Fig DDC injury in *Irs2*^−/−^ mice leads to more rapid activation of tissue-remodeling genes and mobilization of bone-marrow–derived stroma. (A) Early induction of fibrogenic genes in *Irs2*^−/−^ mice coincided with increased leukocyte gene expression. RT-qPCR analysis of whole-liver mRNA using a panel of genes associated with tissue remodeling and bone-marrow–derived stroma (*n* = 6–8). *Irs2*^−/−^ mice displayed increased early induction of profibrogenic cytokine *Tgf*β, transcription factor *Myc*, and tissue-remodeling factors Timp1/Mmp9 on day 7. This coincided with a dramatic peak in myeloid stem cell factor (Kit) and leukocyte gene expression (*Ptprc*/Cd45). (B) Increased Thy1/Cd45 colocalization in DDC livers of *Irs2*^−/−^ mice indicates greater incorporation of bone-marrow–derived cells into the stromal niche. Confocal immunofluorescence images of WT and *Irs2*^−/−^ livers after 21 days of DDC feeding. Rounded Cd45+ cells typical of leukocytes were observed in WT livers (*), whereas Cd45+ cells in *Irs2*^−/−^ livers coexpressed Thy1 and were more flattened (dotted arrows). Selected images are representative of *n* = 4. White dotted line = portal vein. Yellow boxes mark expanded regions of interest. (C) Mobilization of T lymphocytes increased in DDC livers of *Irs2*^−/−^ mice. Immunohistochemical staining for T-cell marker Cd3 on DDC day 21. (Left) Representative images of Cd3 immunostaining highlighting T cells (red arrows). (Right) Graphical quantification of Cd3+ T-cell numbers in WT and Irs2^−/−^ livers (*n* = 6). Data information: underlying data are available in S2 Data. Data are presented as mean + SEM. *P < 0.05, **P < 0.01, and ***P < 0.001. (A) Two-way ANOVA was used to compare means. Significance *P* values were calculated using Tukey's multiple comparison test. (C) Unpaired Student *t* test. DDC, 3.5-diethoxycarbonyl-1.4-dihydrocollidine; *Irs2*, insulin receptor substrate 2; *Kit*, proto-oncogene c-Kit; mRNA, messenger RNA; *Mmp9*, matrix metallopeptidase 9; *Myc*, MYCO proto-oncogene; *Ptprc*, Protein Tyrosine Phosphatase, Receptor Type C gene encoding CD45; RT-qPCR, reverse transcriptase-quantitative PCR; *Tgfβ*, transforming growth factor beta; Thy1, Thy-1 cell surface antigen; *Timp1*, tissue inhibitor of metalloproteinase 1; WT, wild type.
(TIF)

S7 Fig Stable silencing of IRS2 in LX-2 cells had no measurable impact on fibrogenic gene expression or cell viability. (A) Stable knockdown of *IRS2* was performed in LX-2 cells using lentiviral shRNA (sh-IRS2) versus control vector (sh-luc). RT-qPCR was then performed for indicated HSC genes under standard culture conditions (*n* = 3). (B) MTT assay was used to assess cell viability in IRS2 knockdown (sh-IRS2) versus control (sh-luc) LX-2 cells (*n* = 3). Data information: underlying data are available in S2 Data. Data are presented as mean + SEM. *P < 0.05, **P < 0.01, and ***P < 0.001. Paired Student *t* test was used to compare means. HSC, hepatic stellate cell; *Irs2*, insulin receptor substrate 2; MTT, 3-(4,5-dimethylthiazol-2-yl)-2,5-diphenyltetrazolium bromide; RT-qPCR, reverse transcriptase-quantitative PCR; shIRS2, shRNA-targeting IRS2; sh-luc, control luciferase; shRNA, short hairpin RNA.
(TIF)

S8 Fig Differentiation of bipotent human HepaRG to hepatocytes is insulin and *IRS2* dependent. (A) Schematic: bipotent HepaRG cells differentiate to produce “islands” of hepatocyte-like cells. (B, C) Insulin signaling promotes HepaRG–hepatocyte differentiation. (B) Phase-contrast (Phase) and immunofluorescence images of HepaRG cells differentiated in "control" media with insulin supplement (0.88 μM) or in media in which the supplement was excluded (−). Cells stably transduced with a GFP reporter construct driven by the human APOA2 promoter (pAPOA2-GFP) or Albumin/HNF4α immunostaining were used to visualize hepatocyte islands. H = Hoechst. (C) Quantification of p*APOA2*-GFP expression with time during HepaRG differentiation in the presence (control) or absence (ins−) of supplemented insulin (*n* = 3). (D) Stable silencing of IRS2 promotes insulin resistance in HepaRG cells. Above: schematic showing how the IRS2 scaffold protein couples the activated receptor tyrosine kinase to intracellular effectors such as PI3K. Below: western blot showing stable knockdown of IRS2 and concomitant reduction in the activation of PI3K downstream of insulin stimulation, as judged by reduced phosphorylation PI3K effector AKT (Serine 473). (E, F) Stable silencing of IRS2 in HepaRG blocked hepatocyte differentiation in the presence of insulin. (E) Immunofluorescence stainings for hepatocyte markers Albumin, HNF4α, and CYP3A4 of differentiated HepaRG cells following stable lentiviral transduction with control (sh-scram) or sh*IRS2* coexpressing GFP. H = Hoechst. (F) INcell quantification of hepatocyte differentiation (*n* = 3). Data information: underlying data available in S2 Data. Data are presented as mean + SEM. *P < 0.05, **P < 0.01, and ***P < 0.001. (C) Two-way ANOVA was used to compare means. Significance *P* values were calculated using Bonferroni test. (F) Unpaired Student *t* test. AKT, Protein kinase B; *APOA2*, apolipoprotein A2; CYP3A4, cytochrome P450 3A4; GFP, green fluorescent protein; HNF4α, hepatocyte nuclear factor 4-alpha; ins, insulin; *Irs2*, insulin receptor substrate 2; *pAPOA2*, *APOA2* promoter; PI3k, phosphoinositide 3-kinase; shIRS2, shRNA-targeting IRS2; shRNA, short hairpin RNA; sh-scram, scrambled shRNA.
(TIF)

S9 Fig Treatment of HepaRG cultures with rhFGF7 promoted rapid induction of osteopontin/*SPP1* expression in vitro. RT-qPCR time course of rhFGF7 response in HepaRG cells (day 13). Changes in osteopontin/SPP1 are compared to vehicle-treated cont. Data information: underlying data are available in S2 Data. Data are presented as mean + SEM. Data are presented as mean + SEM. *P < 0.05, **P < 0.01, and ***P < 0.001. Two-way ANOVA was used to compare means. Significance *P* values were calculated using Tukey's multiple comparison test. cont, control; Fgf7, fibroblast growth factor 7; rhFGF7, recombinant human FGF7; RT-qPCR, reverse transcriptase-quantitative PCR; Spp1, secreted phosphoprotein 1.
(TIF)

S10 Fig Impact of stromal *IRS2* gene silencing on LX-2/HepaRG cocultures. (A) RT-qPCR time course showing changes in FGFR2-IIIb ligand gene expression in LX-2/HepaRG cocultures. Silencing of *IRS2* in LX-2 cells resulted in impaired *FGF10* induction (above) but had no impact upon *FGF22* expression (below) (*n* = 3). (B) *IRS2* was required for time-dependent switching between *THY1* and *SPP1*. Day 2 and day 14 LX-2/HepaRG cocultures maintained in media with (+) or without (−) supplemented insulin were analyzed for mesenchymal genes associated with myofibroblasts (*THY1*) or LPCs (*SPP1*) by RT-qPCR. Switching from *THY1* to *SPP1* was observed in cocultures using control LX-2 stroma (sh-luc) but not in those in which IRS2 was silenced (sh-*IRS2*) (*n* = 3). (C) Silencing of *IRS2* favored expansion of LX-2 cells in HepaRG coculture. Phase-contrast (Phase) and immunofluorescence images (GFP) taken on day 14 of LX-2/HepaRG coculture. Stromal expansion within the cocultures (s) was tracked by lentiviral coexpression of GFP in control (sh-luc) or *IRS2*-deficient (sh-IRS2) LX-2 cells. (Images are representative of *n* = 3.) Data information: underlying data are available in S2 Data. Data are presented as mean + SEM. *P < 0.05, **P < 0.01, and ***P < 0.001. Two-way ANOVA was used to compare means. Significance *P* values were calculated using (A) Tukey's or (B) Sidak’s multiple comparison tests. Fgfr2-IIIB, Fgf7 receptor; Fgf7, fibroblast growth factor 7; *Irs2*, insulin receptor substrate 2; RT-qPCR, reverse transcriptase-quantitative PCR; shIRS2, shRNA-targeting IRS2; sh-luc, control luciferase; shRNA, short hairpin RNA; Spp1, secreted phosphoprotein 1; Thy1, Thy-1 cell surface antigen.
(TIF)

S1 Table Lentiviral vectors used to generate stable cell lines. (DOCX)

S2 Table List of antibodies and conditions used for western blot, ICC, and IHC. ICC, immunocytochemistry; IHC, immunohistochemistry.
(DOCX)

S3 Table List of primers used for RT-qPCR. RT-qPCR, reverse transcriptase-quantitative PCR.
(DOCX)

S1 Data Underlying data for main figures. (XLSX)

S2 Data Underlying data for supporting figures. (XLSX)

## Abbreviations

AKT: Protein kinase B
ALT: alanine transaminase
APOA2: apolipoprotein A2
AST: aspartate transaminase
AU: arbitrary unit
c-CASP3: cleaved caspase 3
CEEA: Comite Ético de Experimentación Animal
cMyc: MYC proto-oncogene
COL1A1: Collagen 1A1
COL3A1: type III collagen
Ctgf: Connective tissue growth factor
CYP3A4: cytochrome P450 3A4
db/db: diabetic leptin-deficient mice
DDC: 3.5-diethoxycarboxynl-1.4-dihydrocollidine
DR: ductular reaction
E-cad: E-cadherin
EF1: Human elongation factor-1 alpha promoter
EpCAM: epithelial cell adhesion molecule
ERK: extracellular signal-regulated kinase
Fc: Fragment crystallizable region
Fgfr2-IIIb: Fibroblast growth factor receptor 2 isoform IIIb
Fgf7: fibroblast growth factor 7
GAPDH: Glyceraldehyde-3-Phosphate Dehydrogenase
Gfap: glial fibrillary acidic protein
GFP: green fluorescent protein
hHSC: human HSC
HNF4α: hepatocyte nuclear factor 4-alpha
HSC: hepatic stellate cell
HSPA1B: heat shock 70 kDa protein 1B
IGF-1: Insulin-like growth factor 1
i.p.: intraperitoneal
Kit: Proto-oncogene c-kit
Irs2: insulin receptor substrate 2
Lenti-mIrs2: murine *Irs2* transgene not targeted by human-gene–silencing construct
LPC: liver progenitor cell; MitoC, mitomycin C
Mmp9: Matrix metallopeptidase 9
mRNA: messenger RNA
MTT: 3-(4,5-dimethylthiazol-2-yl)-2,5-diphenyltetrazolium bromide
Myc: MYCO proto-oncogene
NAFLD: nonalcoholic fatty liver disease
NASH: nonalcoholic steatohepatitis
NPC: nonparenchymal cell
NT-157: allosteric inhibitor of IRS proteins 1 and 2
OD: optical density
pAPOA2: *APOA2* promoter
P-Erk: phosphorylated Erk (Tyrosine 204)
PF: portal fibroblast
pHSC: primary human HSC
pIRS2: *Irs2* promoter
PI3K: phosphoinositide 3-kinase
Ptprc: protein tyrosine phosphatase, receptor type C gene encoding CD45 cell surface antigen
rhFGFR2-IIIb/rhIIIb-Fc: recombinant human Fgfr2
rFgf7: recombinant Fgf7
rhFGF7: recombinant human Fgf7
RPL19: Ribosomal Protein L19
RT-qPCR: reverse transcriptase-quantitative PCR
RU: relative unit
shIRS2: shRNA-targeting IRS2
sh-luc: control luciferase
shRNA: short hairpin RNA
SPF: specific-pathogen–free
Spp1: secreted phosphoprotein 1
TAZ: Tafazzin
TBIL: total bilirubin
Tgfβ: transforming growth factor beta
Thy1: Thy-1 cell surface antigen
Timp1: tissue inhibitor of metalloproteinase 1
WT: wild type
YAP: Yes-associated protein
αSma/Acta2: alpha-smooth muscle actin

## References

[1] Werner S, Grose R. Regulation of wound healing by growth factors and cytokines. Physiol Rev. 2003;83: 835–870. 10.1152/physrev.2003.83.3.835 12843410

[2] Brem H, Tomic-Canic M. Cellular and molecular basis of wound healing in diabetes. J Clin Invest. 2007;117: 1219–22. 10.1172/JCI32169 17476353 PMC1857239

[3] Byrne CD, Targher G. NAFLD: A multisystem disease. Journal of Hepatology. 2015;62:S47–S64. 10.1016/j.jhep.2014.12.012 25920090

[4] Katsumata LW, Miyajima A, Itoh T. Portal fibroblasts marked by the surface antigen Thy1 contribute to fibrosis in mouse models of cholestatic liver injury. Hepatol Commun. 2017;1: 198–214. 10.1002/hep4.1023 29404454 PMC5721447

[5] Leclercq IA, Da Silva Morais A, Schroyen B, Van Hul N, Geerts A. Insulin resistance in hepatocytes and sinusoidal liver cells: mechanisms and consequences. J Hepatol. 2007;47: 142–56. 10.1016/j.jhep.2007.04.002 17512085

[6] Withers DJ, Gutierrez JS, Towery H, Burks DJ, Ren JM, Previs S, et al Disruption of IRS-2 causes type 2 diabetes in mice. Nature. 1998;391: 900–904. 10.1038/36116 9495343

[7] Valverde AM, Burks DJ, Fabregat I, Fisher TL, Carretero J, White MF, et al Molecular Mechanisms of Insulin Resistance in IRS-2-Deficient Hepatocytes. Diabetes. 2003;52:2239–2248. 12941762 10.2337/diabetes.52.9.2239

[8] Kubota N, Kubota T, Kajiwara E, Iwamura T, Kumagai H, Watanabe T, et al Differential hepatic distribution of insulin receptor substrates causes selective insulin resistance in diabetes and obesity. Nat Commun. 2016;7: 12977 10.1038/ncomms12977 27708333 PMC5059684

[9] Kubota N, Kubota T, Itoh S, Kumagai H, Kozono H, Takamoto I, et al Dynamic Functional Relay between Insulin Receptor Substrate 1 and 2 in Hepatic Insulin Signaling during Fasting and Feeding. Cell Metab. 2008;8: 49–64. 10.1016/j.cmet.2008.05.007 18590692

[10] Rametta R, Mozzi E, Dongiovanni P, Motta BM, Milano M, Roviaro G, et al Increased insulin receptor substrate 2 expression is associated with steatohepatitis and altered lipid metabolism in obese subjects. Int J Obes. 2013;37: 986–992. 10.1038/ijo.2012.181 23147115

[11] Kawaguchi T, Yoshida T, Harada M, Hisamoto T, Nagao Y, Ide T, et al Hepatitis C virus down-regulates insulin receptor substrates 1 and 2 through up-regulation of suppressor of cytokine signaling 3. Am J Pathol. 2004;165: 1499–508. 10.1016/S0002-9440(10)63408-6 15509521 PMC1618659

[12] Boissan M, Beurel E, Wendum D, Rey C, Lécluse Y, Housset C, et al Overexpression of insulin receptor substrate-2 in human and murine hepatocellular carcinoma. Am J Pathol. American Society for Investigative Pathology; 2005;167: 869–77. 10.1016/S0002-9440(10)62058-5 16127164 PMC1698721

[13] Jeong S-H, Kim H-B, Kim M-C, Lee J, Lee JH, Kim J-H, et al Hippo-mediated suppression of IRS2/AKT signaling prevents hepatic steatosis and liver cancer. J Clin Invest. 2018;128: 1010–1025. 10.1172/JCI95802 29400692 PMC5824861

[14] Lee K-P, Lee J-H, Kim T-S, Kim T-H, Park H-D, Byun J-S, et al The Hippo-Salvador pathway restrains hepatic oval cell proliferation, liver size, and liver tumorigenesis. Proc Natl Acad Sci U S A. 2010;107: 8248–8253. 10.1073/pnas.0912203107 20404163 PMC2889558

[15] Steinberg Z, Myers C, Heim VM, Lathrop CA, Rebustini IT, Stewart JS, et al FGFR2b signaling regulates ex vivo submandibular gland epithelial cell proliferation and branching morphogenesis. Development. 2005;132: 1223–1234. 10.1242/dev.01690 15716343

[16] Iseki S, Wilkie AO, Morriss-Kay GM. Fgfr1 and Fgfr2 have distinct differentiation- and proliferation-related roles in the developing mouse skull vault. Development. 1999;126: 5611–20. Available from: http://www.ncbi.nlm.nih.gov/pubmed/10572038 10572038 10.1242/dev.126.24.5611

[17] Werner S, Smola H, Liao X, Longaker MT, Krieg T, Hofschneider PH, et al The function of KGF in morphogenesis of epithelium and reepithelialization of wounds. Science. 1994;266: 819–22. Available from: http://www.ncbi.nlm.nih.gov/pubmed/7973639 7973639 10.1126/science.7973639

[18] Steiling H, Mühlbauer M, Bataille F, Schölmerich J, Werner S, Hellerbrand C. Activated hepatic stellate cells express keratinocyte growth factor in chronic liver disease. Am J Pathol. 2004;165: 1233–1241. 10.1016/S0002-9440(10)63383-4 15466389 PMC1618645

[19] Takase HM, Itoh T, Ino S, Wang T, Koji T, Akira S, et al FGF7 is a functional niche signal required for stimulation of adult liver progenitor cells that support liver regeneration. Genes Dev. 2013;27: 169–181. 10.1101/gad.204776.112 23322300 PMC3566310

[20] Kaneko K, Kamimoto K, Miyajima A, Itoh T. Adaptive remodeling of the biliary architecture underlies liver homeostasis. Hepatology. 2015;61: 2056–2066. 10.1002/hep.27685 25572923

[21] Itoh N, Nakayama Y, Konishi M. Roles of FGFs As Paracrine or Endocrine Signals in Liver Development, Health, and Disease. Front Cell Dev Biol. 2016;4: 1–9. 10.3389/fcell.2016.00001 27148532 PMC4829580

[22] Utley S, James D, Mavila N, Nguyen M V., Vendryes C, Salisbury SM, et al Fibroblast growth factor signaling regulates the expansion of A6-expressing hepatocytes in association with AKT-dependent β-catenin activation. J Hepatol. 2014;60: 1002–1009. 10.1016/j.jhep.2013.12.017 24365171 PMC3995894

[23] Turner N, Grose R. Fibroblast growth factor signaling: from development to cancer. Nat Rev Cancer. 2010;10: 116–129. 10.1038/nrc2780 20094046

[24] Chell JM, Brand AH. Nutrition-responsive glia control exit of neural stem cells from quiescence. Cell. 2010;143: 1161–1173. 10.1016/j.cell.2010.12.007 21183078 PMC3087489

[25] Cheetham SW, Brand AH. Cell biology. Insulin finds its niche. Science. 2013;340: 817–8. 10.1126/science.1238525 23687033

[26] Wang B, Zhao L, Fish M, Logan CY, Nusse R. Self-renewing diploid Axin2 + cells fuel homeostatic renewal of the liver. Nature. 2015;524: 180–185. 10.1038/nature14863 26245375 PMC4589224

[27] Font-Burgada J, Shalapour S, Ramaswamy S, Hsueh B, Rossell D, Umemura A, et al Hybrid Periportal Hepatocytes Regenerate the Injured Liver without Giving Rise to Cancer. Cell. 2015;162: 766–779. 10.1016/j.cell.2015.07.026 26276631 PMC4545590

[28] Xu J, Kisseleva T. Bone marrow-derived fibrocytes contribute to liver fibrosis. Exp Biol Med. 2015;240: 691–700. 10.1177/1535370215584933 25966982 PMC4866973

[29] Reuveni H, Flashner-Abramson E. Therapeutic destruction of insulin receptor substrates for cancer treatment. Cancer Res. 2013;73: 4383–4394. 10.1158/0008-5472.CAN-12-3385 23651636 PMC4391644

[30] Parent R, Marion M-J, Furio L, Trépo C, Petit M-A. Origin and characterization of a human bipotent liver progenitor cell line. Gastroenterology. 2004;126: 1147–56. Available from: http://www.ncbi.nlm.nih.gov/pubmed/15057753 15057753 10.1053/j.gastro.2004.01.002

[31] Kisseleva T, Cong M, Paik Y, Scholten D, Jiang C, Benner C, et al Myofibroblasts revert to an inactive phenotype during regression of liver fibrosis. Proc Natl Acad Sci. 2012;109: 9448–9453. 10.1073/pnas.1201840109 22566629 PMC3386114

[32] Richardson MM, Jonsson JR, Powell EE, Brunt EM, Neuschwander–Tetri BA, Bhathal PS, et al Progressive Fibrosis in Nonalcoholic Steatohepatitis: Association With Altered Regeneration and a Ductular Reaction. Gastroenterology. Elsevier; 2007;133: 80–90. 10.1053/j.gastro.2007.05.012 17631134

[33] Fickert P, Stöger U, Fuchsbichler A, Moustafa T, Marschall H-U, Weiglein AH, et al A new xenobiotic-induced mouse model of sclerosing cholangitis and biliary fibrosis. Am J Pathol. American Society for Investigative Pathology; 2007;171: 525–36. 10.2353/ajpath.2007.061133 17600122 PMC1934539

[34] Svegliati-Baroni G, Faraci G, Fabris L, Saccomanno S, Cadamuro M, Pierantonelli I, et al Insulin resistance and necroinflammation drives ductular reaction and epithelial-mesenchymal transition in chronic hepatitis C. Gut. BMJ Publishing Group; 2011;60: 108–15. 10.1136/gut.2010.219741 20966027 PMC3784835

[35] Sanchez-Lopez E, Flashner-Abramson E, Shalapour S, Zhong Z, Taniguchi K, Levitzki A, et al Targeting colorectal cancer via its microenvironment by inhibiting IGF-1 receptor-insulin receptor substrate and STAT3 signaling. Oncogene. 2016;35: 2634–2644. 10.1038/onc.2015.326 26364612 PMC4791217

[36] Yimlamai D, Christodoulou C, Galli GG, Yanger K, Pepe-Mooney B, Gurung B, et al Hippo pathway activity influences liver cell fate. Cell. Cell Press; 2014;157: 1324–1338. 10.1016/j.cell.2014.03.060 24906150 PMC4136468

[37] Zhou D, Conrad C, Xia F, Park JS, Payer B, Yin Y, et al Mst1 and Mst2 Maintain Hepatocyte Quiescence and Suppress Hepatocellular Carcinoma Development through Inactivation of the Yap1 Oncogene. Cancer Cell. 2009;16: 425–438. 10.1016/j.ccr.2009.09.026 19878874 PMC3023165

[38] Lu L, Finegold MJ, Johnson RL. Hippo pathway coactivators Yap and Taz are required to coordinate mammalian liver regeneration. Exp Mol Med. 2018;50: e423 10.1038/emm.2017.205 29303509 PMC5992983

[39] Yan G, Fukabori Y, McBride G, Nikolaropolous S, McKeehan WL. Exon switching and activation of stromal and embryonic fibroblast growth factor (FGF)-FGF receptor genes in prostate epithelial cells accompany stromal independence and malignancy. Mol Cell Biol. 1993;13: 4513–4522. 10.1128/MCB.13.8.4513.Updated 7687739 PMC360063

[40] Berg T, Rountree CB, Lee L, Estrada J, Sala FG, Choe A, et al Fibroblast growth factor 10 is critical for liver growth during embryogenesis and controls hepatoblast survival via β-catenin activation. Hepatology. 2007;46: 1187–1197. 10.1002/hep.21814 17668871 PMC3494299

[41] Nies VJM, Sancar G, Liu W, van Zutphen T, Struik D, Yu RT, et al Fibroblast Growth Factor Signaling in Metabolic Regulation. Front Endocrinol (Lausanne). Frontiers Media SA; 2015;6: 193 10.3389/fendo.2015.00193 26834701 PMC4718082

[42] Steiling H, Wüstefeld T, Bugnon P, Brauchle M, Fässler R, Teupser D, et al Fibroblast growth factor receptor signaling is crucial for liver homeostasis and regeneration. Oncogene. Nature Publishing Group; 2003;22: 4380–4388. 10.1038/sj.onc.1206499 12853974

[43] Böhm F, Speicher T, Hellerbrand C, Dickson C, Partanen JM, Ornitz DM, et al FGF receptors 1 and 2 control chemically induced injury and compound detoxification in regenerating livers of mice. Gastroenterology. 2010;139: 1385–1396. 10.1053/j.gastro.2010.06.069 20603121 PMC2949525

[44] Padrissa-Altés S, Bachofner M, Bogorad RL, Pohlmeier L, Rossolini T, Böhm F, et al Control of hepatocyte proliferation and survival by Fgf receptors is essential for liver regeneration in mice. Gut. 2015;64: 1444–1453. 10.1136/gutjnl-2014-307874 25416068

[45] Werner S, Breeden M, Hübner G, Greenhalgh DG, Longaker MT. Induction of keratinocyte growth factor expression is reduced and delayed during wound healing in the genetically diabetic mouse. J Invest Dermatol. 1994;103: 469–73. 10.1111/1523-1747.ep12395564 7930669

[46] Peng C, Chen B, Kao H-K, Murphy G, Orgill DP, Guo L. Lack of FGF-7 Further Delays Cutaneous Wound Healing in Diabetic Mice. Plast Reconstr Surg. 2011;128: 673e–684e. 10.1097/PRS.0b013e318230c521 22094769

[47] Walker A, Nissen E, Geiger A. Migratory, metabolic and functional alterations of fibrocytes in type 2 diabetes. IUBMB Life. 2018;70: 1122–1132. 10.1002/iub.1920 30184318

[48] Beckonert O, Keun HC, Ebbels TMD, Bundy J, Holmes E, Lindon JC, et al Metabolic profiling, metabolomic and metabonomic procedures for NMR spectroscopy of urine, plasma, serum and tissue extracts. Nat Protoc. 2007;2: 2692–2703. 10.1038/nprot.2007.376 18007604

[49] Wishart DS, Feunang YD, Marcu A, Guo AC, Liang K, Vázquez-Fresno R, et al HMDB 4.0: The human metabolome database for 2018. Nucleic Acids Res. 2018;46: D608–D617. 10.1093/nar/gkx1089 29140435 PMC5753273

[50] Ulrich EL, Akutsu H, Doreleijers JF, Harano Y, Ioannidis YE, Lin J, et al BioMagResBank. Nucleic Acids Res. 2008;36: D402–D408. 10.1093/nar/gkm957 17984079 PMC2238925

[51] Weiskirchen R, Gressner AM. Isolation and culture of hepatic stellate cells. Methods Mol Med. 2005;117: 99–113. 10.1385/1-59259-940-0:099 16118448

