# Supplementary figures and images for: Insulin resistance disrupts epithelial repair and niche-progenitor Fgf signaling during chronic liver injury
Source: PLoS Biol. 2019 Jan 29;17(1):e2006972. doi: 10.1371/journal.pbio.2006972 (PMC6368328; doi:10.1371/journal.pbio.2006972)

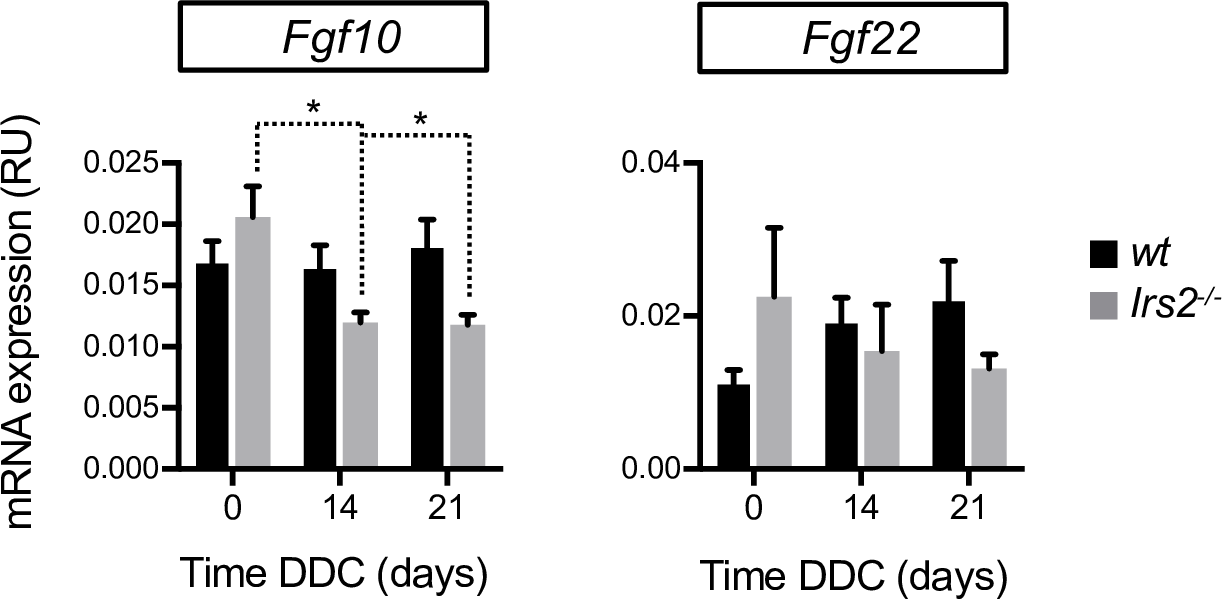

Supplement: S1 Fig — Whole-liver mRNA levels were assessed for Fgf7 family member genes Fgf10 and Fgf22 in WT and Irs2−/− mice during a time course of DDC feeding by RT-qPCR (n = 3–8). Data information: underlying data are available in S2 Data. Data are represented as mean + SEM: Two-way ANOVA was used to compare means. Significance P values were calculated using Tukey’s multiple comparison test (*P < 0.05). Dotted lines indicate statistically significant decrease with time. DDC, 3.5-diethoxycarbonyl-1.4-dihydrocollidine; Fgfr2-IIIB, Fgf7 receptor; Fgf7, fibroblast growth factor 7; Irs2, insulin receptor substrate 2; mRNA, messenger RNA; RT-qPCR, reverse transcriptase-quantitative PCR; WT, wild type. (TIF) [file pbio.2006972.s001.tif]

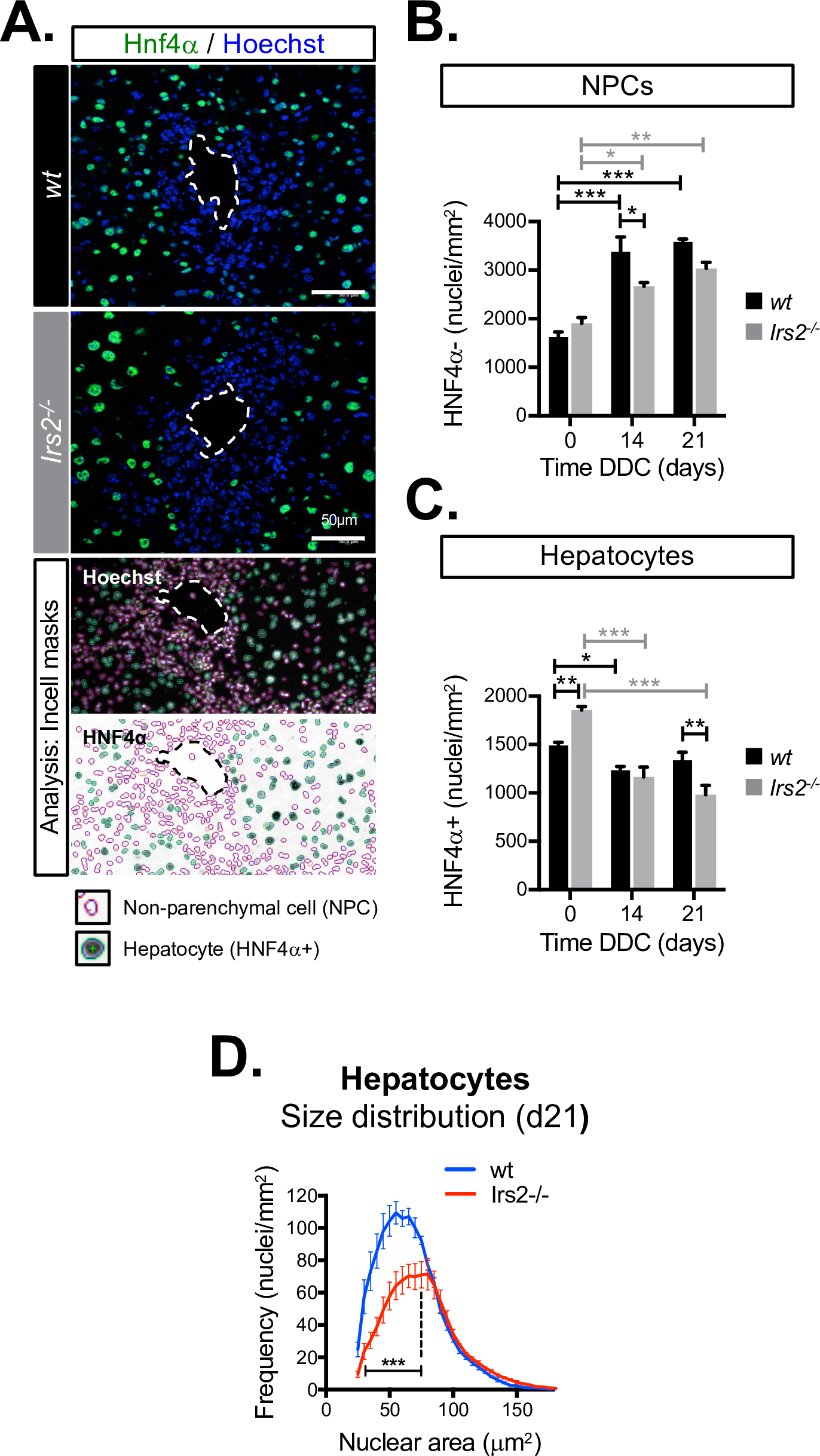

Supplement: S2 Fig — (A–C) HNF4α immunostaining was used to quantify the parenchymal (HNF4α+) and NPC (HNF4α−) responses to DDC liver injury in WT and Irs2−/− mice. (A) Representative images of HNF4α immunostaining (DDC day 21). Below: masks used to gate and quantify hepatocyte and NPC densities in whole-liver sections using INcell Analyzer (total cells analyzed: 1.23 × 10^6^). (B) Time course of DDC liver injury comparing the numbers of NPC nuclei (HNF4α−) in livers of WT and Irs2−/− mice (n = 4–6, total of 7.2 × 10^5^ HNF4α− nuclei analyzed). (C) Time course of DDC liver injury comparing the numbers of hepatocyte nuclei (HNF4α+) in livers of WT and Irs2−/− mice (n = 4–6, total of 5.1 × 10^5^ HNF4α+ nuclei analyzed). (D) Size distribution of HNF4α+ hepatocyte nuclei in livers of WT and Irs2−/− mice following DDC liver injury (d21), calculated in situ using INCell Analyzer. Data show significant depletion of small hepatocytes nuclear area < 75 μm2. (n = 4, total of 1.2 × 10^5^ HNF4α+ nuclei analyzed). Data information: underlying data are available in S2 Data. Data are presented as mean + SEM. *P < 0.05, **P < 0.01, and ***P < 0.001. Two-way ANOVA was used to compare means. Significance P values were calculated using Bonferroni test. DDC, 3.5-diethoxycarbonyl-1.4-dihydrocollidine; HNF4α, hepatocyte nuclear factor 4-alpha; Irs2, insulin receptor substrate 2; NPC, nonparenchymal cell; WT, wild type. (TIF) [file pbio.2006972.s002.tif]

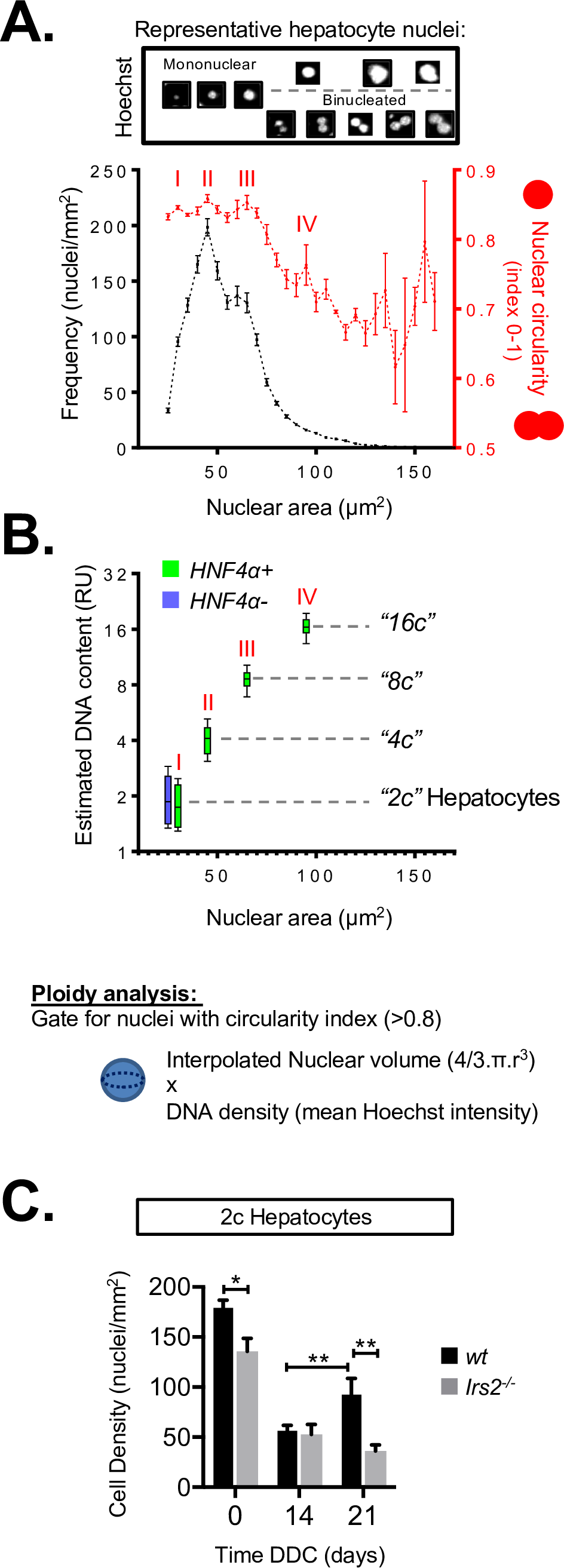

Supplement: S3 Fig — (A–B) Analysis of hepatocyte ploidy in HNF4α immunolabelled liver sections. (A) High-content imaging was used to generate frequency, size, and morphometry profiles of HNF4α+ nuclei based on quantification of Hoechst DNA staining in liver tissue sections. Upper panel shows representative images of hepatocyte nuclei of different sizes and shapes, with smaller nuclei (<75 μm2) tending to have greater circularity indices (>0.8), whereas larger nuclei tended to be bilobular. We observed discrete peaks in nuclear “circularity” (I–IV) that we hypothesized corresponded to the major ploidy groupings in the liver: 2n, 4n, 8n, and 16n. Data shown from untreated WT livers (n = 4, 1.35 × 10^4^ HNF4α+ nuclei per animal). (B) To test this hypothesis, all nuclei were gated for circularity (>0.8), and DNA content was calculated for peaks I–V as a function of interpolated nuclear volume and Hoechst intensity (formula below). Using HNF4α− NPCs as an internal 2n control, we confirmed that populations I–IV accurately represented 2c, 4c, 8c, and 16c hepatocyte populations, respectively (n = 4, 1.1 × 10^4^ HNF4α+ nuclei per animal). This original methodology to describe hepatocyte ploidy in situ was then applied to WT and Irs2−/− livers during DDC feeding. (C) Quantification of small hepatocytes with an estimated 2n DNA content (2c) as calculated in situ using INCell Analyzer showing time-dependent increase in WT livers (days 14–21) and significant depletion in livers of Irs2−/− mice following DDC feeding (day 21) (n = 4–6, total of 4.8 × 10^4^ HNF4α+ nuclei analyzed). Data information: underlying data are available in S2 Data. Data are presented as mean + SEM. *P < 0.05, **P < 0.01, and ***P < 0.001. Two-way ANOVA was used to compare means. Significance P values were calculated using Bonferroni test. DDC, 3.5-diethoxycarbonyl-1.4-dihydrocollidine; HNF4α, hepatocyte nuclear factor 4-alpha; Irs2, insulin receptor substrate 2; NPC, nonparenchymal cell; WT, wild type. (TIF) [file pbio.2006972.s003.tif]

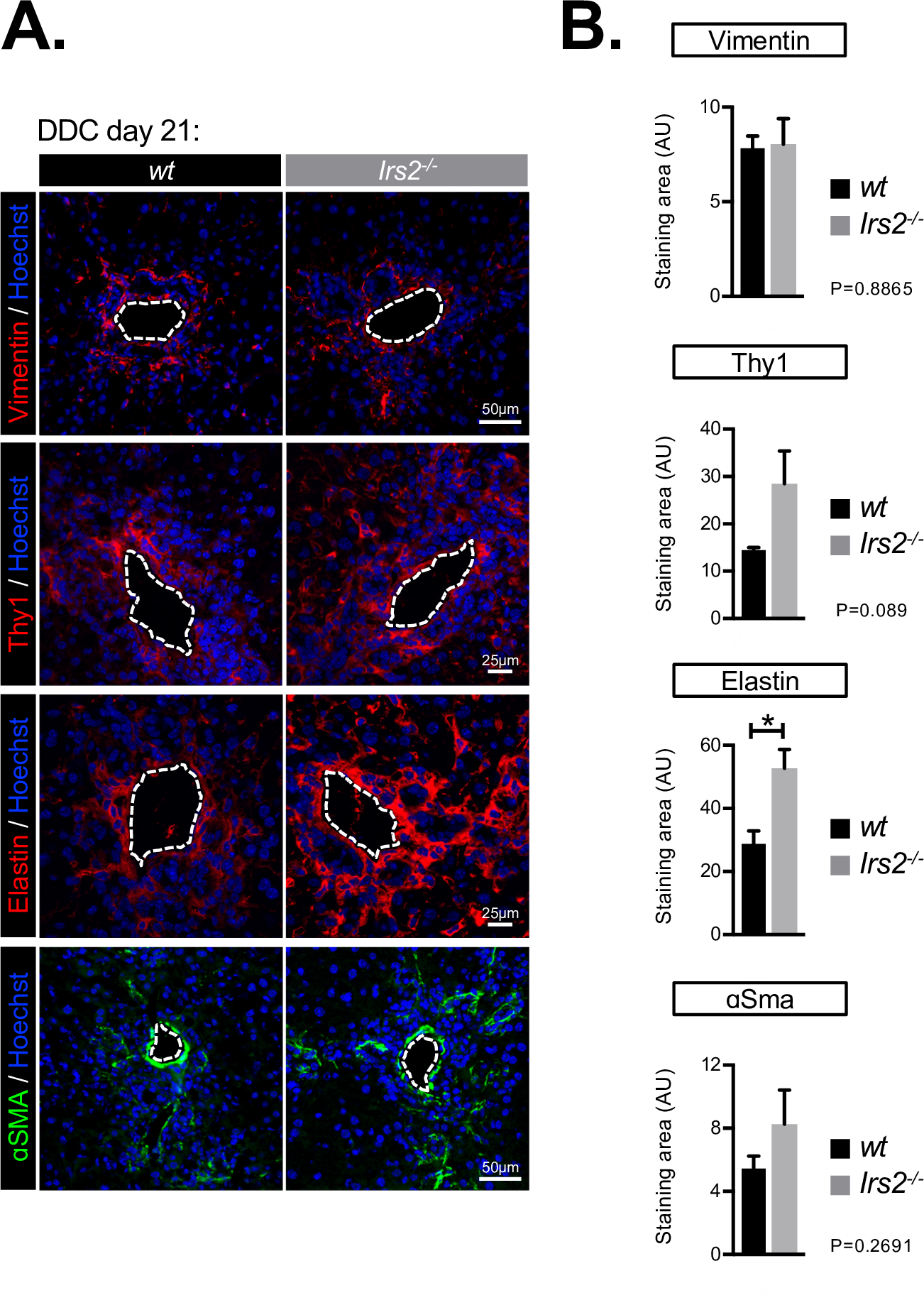

Supplement: S4 Fig — (A–B) Analysis of PF/myofibroblast markers by immunostaining in periportal sections of WT and Irs2−/− mice on day 21 of DDC feeding. (A) Representative confocal images of DDC livers using indicated antibodies. Dotted line = portal vein. (B) Graphical quantification of immunostainings by analysis of staining area (n = 3–4). Data information: underlying data are available in S2 Data. Data are presented as mean + SEM. *P < 0.05. (B) Unpaired Student t test was used to compare means. DDC, 3.5-diethoxycarbonyl-1.4-dihydrocollidine; Irs2, insulin receptor substrate 2; PF, portal fibroblast; WT, wild type. (TIF) [file pbio.2006972.s004.tif]

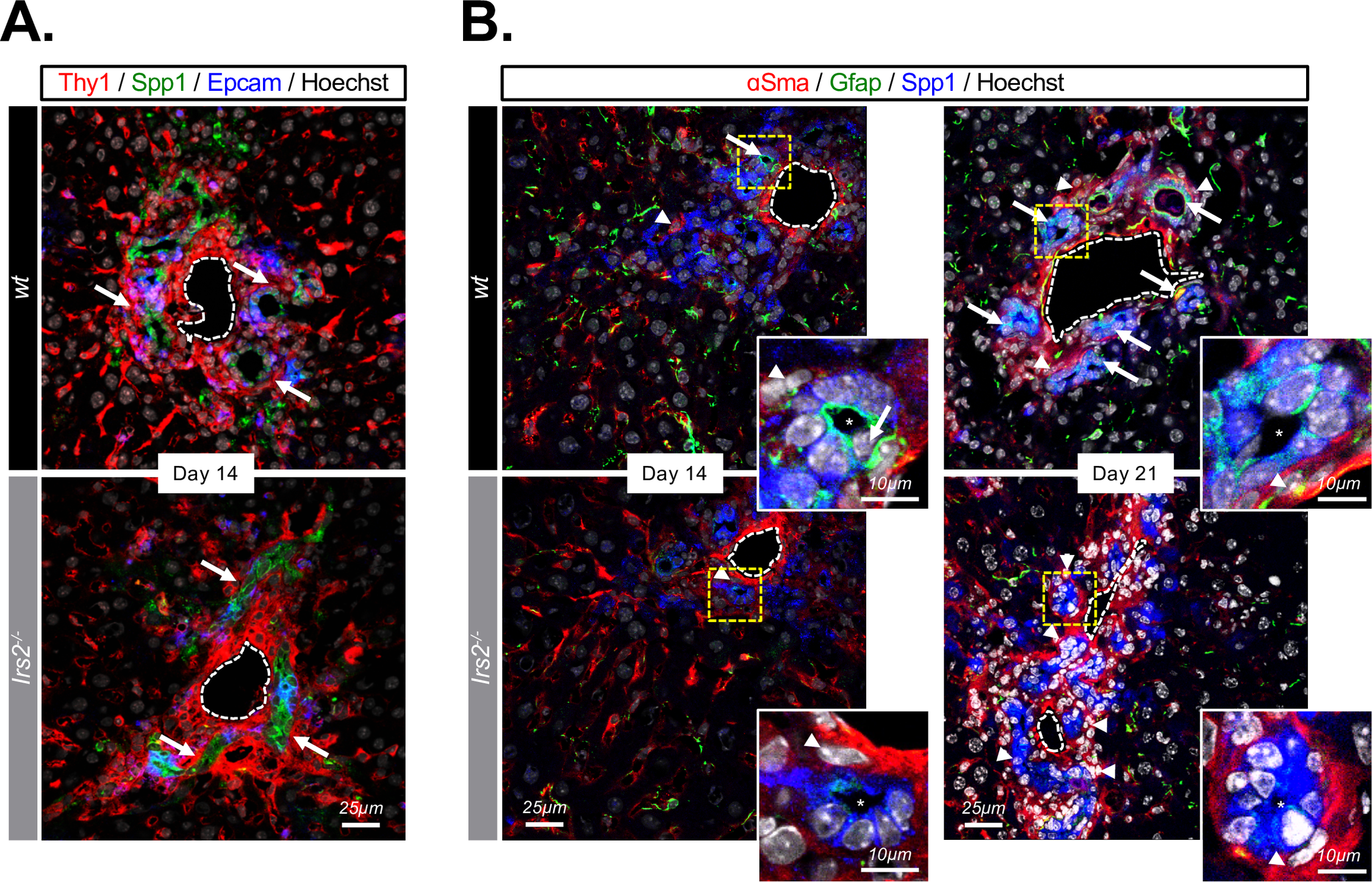

Supplement: S5 Fig — (A–B) Confocal images of immunofluorescence-stained DDC livers describing the stromal environment surrounding LPCs in WT and Irs2−/− mice at the indicated time points. White dotted line = portal vein. (A) Thy1+ cells surrounded ducts containing Epcam+/Spp1+ LPCs in the livers of both WT and Irs2−/− mice (indicated by arrows). Selected images are representative of n = 5. (B) The stromal niche in both WT and Irs2−/− mice also contained αSma+ myofibroblasts (arrowheads) surrounding Spp1+ LPCs. However, Gfap+ HSCs were reduced in number in the Irs2−/− stroma, and contact between Gfap+ cells and LPCs (arrows) was reduced. Yellow dotted boxes mark expanded regions of interest containing representative duct-like structures (*). Selected images are representative of n = 3–5. DDC, 3.5-diethoxycarbonyl-1.4-dihydrocollidine; EpCAM, epithelial cell adhesion molecule; Gfap, glial fibrillary acidic protein; HSC, hepatic stellate cell; Irs2, insulin receptor substrate 2; LPC, liver progenitor cell; PF, portal fibroblast; Spp1, secreted phosphoprotein 1; Thy1, Thy-1 cell surface antigen; WT, wild type; αSma, alpha-smooth actin muscle. (TIF) [file pbio.2006972.s005.tif]

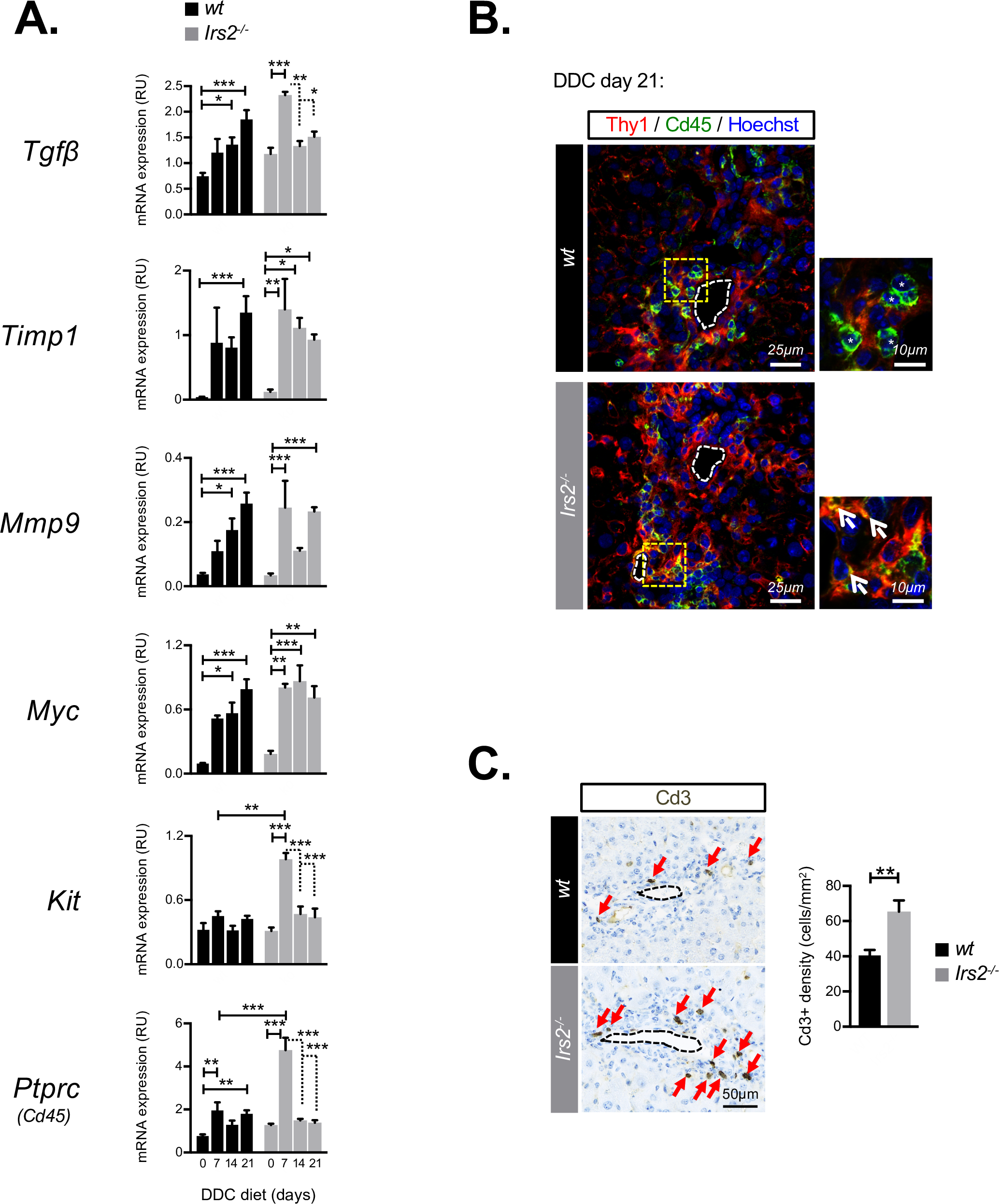

Supplement: S6 Fig — (A) Early induction of fibrogenic genes in Irs2−/− mice coincided with increased leukocyte gene expression. RT-qPCR analysis of whole-liver mRNA using a panel of genes associated with tissue remodeling and bone-marrow–derived stroma (n = 6–8). Irs2−/− mice displayed increased early induction of profibrogenic cytokine Tgfβ, transcription factor Myc, and tissue-remodeling factors Timp1/Mmp9 on day 7. This coincided with a dramatic peak in myeloid stem cell factor (Kit) and leukocyte gene expression (Ptprc/Cd45). (B) Increased Thy1/Cd45 colocalization in DDC livers of Irs2−/− mice indicates greater incorporation of bone-marrow–derived cells into the stromal niche. Confocal immunofluorescence images of WT and Irs2−/− livers after 21 days of DDC feeding. Rounded Cd45+ cells typical of leukocytes were observed in WT livers (*), whereas Cd45+ cells in Irs2−/− livers coexpressed Thy1 and were more flattened (dotted arrows). Selected images are representative of n = 4. White dotted line = portal vein. Yellow boxes mark expanded regions of interest. (C) Mobilization of T lymphocytes increased in DDC livers of Irs2−/− mice. Immunohistochemical staining for T-cell marker Cd3 on DDC day 21. (Left) Representative images of Cd3 immunostaining highlighting T cells (red arrows). (Right) Graphical quantification of Cd3+ T-cell numbers in WT and Irs2−/− livers (n = 6). Data information: underlying data are available in S2 Data. Data are presented as mean + SEM. *P < 0.05, **P < 0.01, and ***P < 0.001. (A) Two-way ANOVA was used to compare means. Significance P values were calculated using Tukey's multiple comparison test. (C) Unpaired Student t test. DDC, 3.5-diethoxycarbonyl-1.4-dihydrocollidine; Irs2, insulin receptor substrate 2; Kit, proto-oncogene c-Kit; mRNA, messenger RNA; Mmp9, matrix metallopeptidase 9; Myc, MYCO proto-oncogene; Ptprc, Protein Tyrosine Phosphatase, Receptor Type C gene encoding CD45; RT-qPCR, reverse transcriptase-quantitative PCR; Tgfβ, transforming growth f [file pbio.2006972.s006.tif]

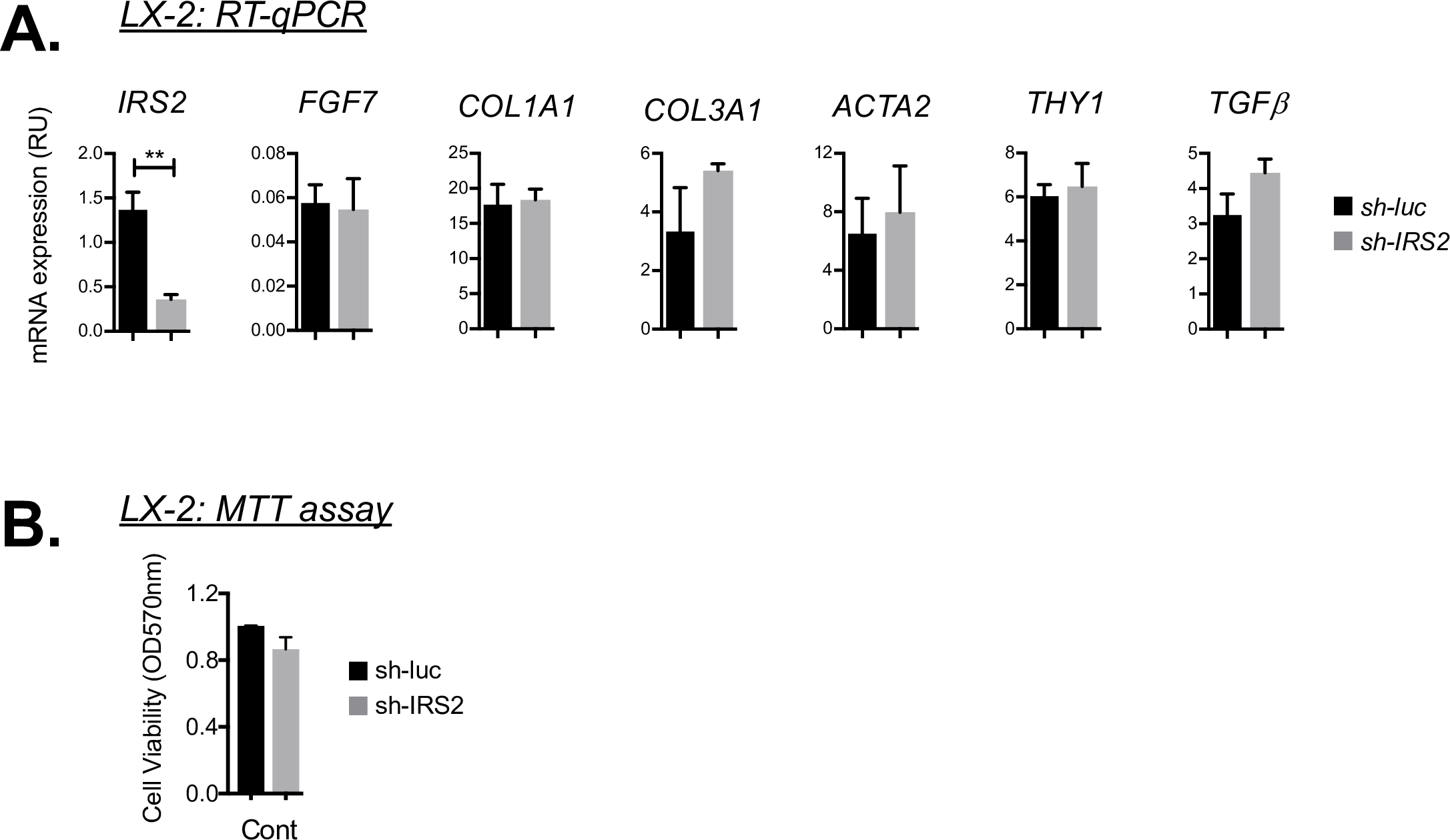

Supplement: S7 Fig — (A) Stable knockdown of IRS2 was performed in LX-2 cells using lentiviral shRNA (sh-IRS2) versus control vector (sh-luc). RT-qPCR was then performed for indicated HSC genes under standard culture conditions (n = 3). (B) MTT assay was used to assess cell viability in IRS2 knockdown (sh-IRS2) versus control (sh-luc) LX-2 cells (n = 3). Data information: underlying data are available in S2 Data. Data are presented as mean + SEM. *P < 0.05, **P < 0.01, and ***P < 0.001. Paired Student t test was used to compare means. HSC, hepatic stellate cell; Irs2, insulin receptor substrate 2; MTT, 3-(4,5-dimethylthiazol-2-yl)-2,5-diphenyltetrazolium bromide; RT-qPCR, reverse transcriptase-quantitative PCR; shIRS2, shRNA-targeting IRS2; sh-luc, control luciferase; shRNA, short hairpin RNA. (TIF) [file pbio.2006972.s007.tif]

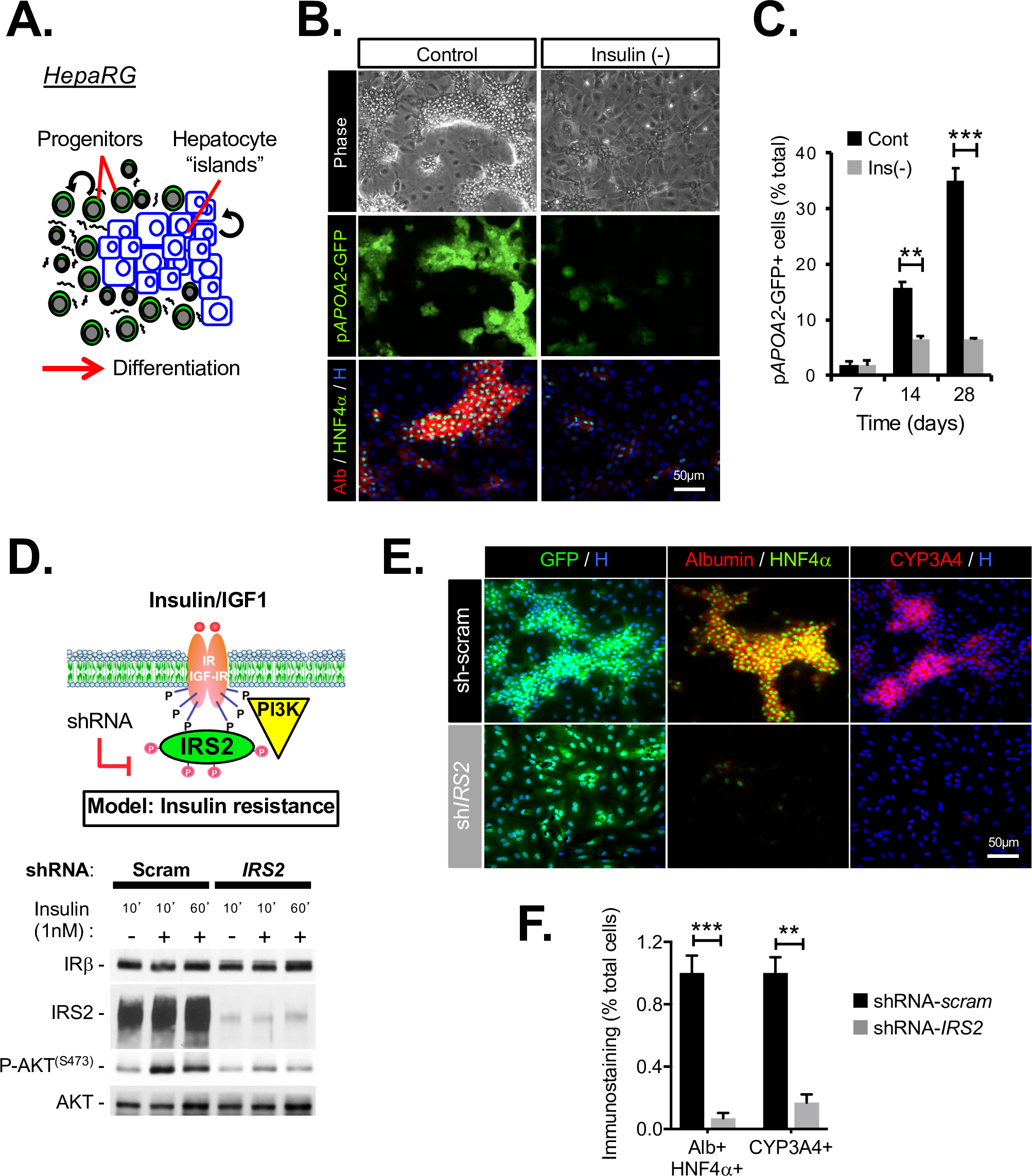

Supplement: S8 Fig — (A) Schematic: bipotent HepaRG cells differentiate to produce “islands” of hepatocyte-like cells. (B, C) Insulin signaling promotes HepaRG–hepatocyte differentiation. (B) Phase-contrast (Phase) and immunofluorescence images of HepaRG cells differentiated in "control" media with insulin supplement (0.88 μM) or in media in which the supplement was excluded (−). Cells stably transduced with a GFP reporter construct driven by the human APOA2 promoter (pAPOA2-GFP) or Albumin/HNF4α immunostaining were used to visualize hepatocyte islands. H = Hoechst. (C) Quantification of pAPOA2-GFP expression with time during HepaRG differentiation in the presence (control) or absence (ins−) of supplemented insulin (n = 3). (D) Stable silencing of IRS2 promotes insulin resistance in HepaRG cells. Above: schematic showing how the IRS2 scaffold protein couples the activated receptor tyrosine kinase to intracellular effectors such as PI3K. Below: western blot showing stable knockdown of IRS2 and concomitant reduction in the activation of PI3K downstream of insulin stimulation, as judged by reduced phosphorylation PI3K effector AKT (Serine 473). (E, F) Stable silencing of IRS2 in HepaRG blocked hepatocyte differentiation in the presence of insulin. (E) Immunofluorescence stainings for hepatocyte markers Albumin, HNF4α, and CYP3A4 of differentiated HepaRG cells following stable lentiviral transduction with control (sh-scram) or shIRS2 coexpressing GFP. H = Hoechst. (F) INcell quantification of hepatocyte differentiation (n = 3). Data information: underlying data available in S2 Data. Data are presented as mean + SEM. *P < 0.05, **P < 0.01, and ***P < 0.001. (C) Two-way ANOVA was used to compare means. Significance P values were calculated using Bonferroni test. (F) Unpaired Student t test. AKT, Protein kinase B; APOA2, apolipoprotein A2; CYP3A4, cytochrome P450 3A4; GFP, green fluorescent protein; HNF4α, hepatocyte nuclear factor 4-alpha; ins, insulin; Irs2, insulin receptor substrate 2; pAP [file pbio.2006972.s008.tif]

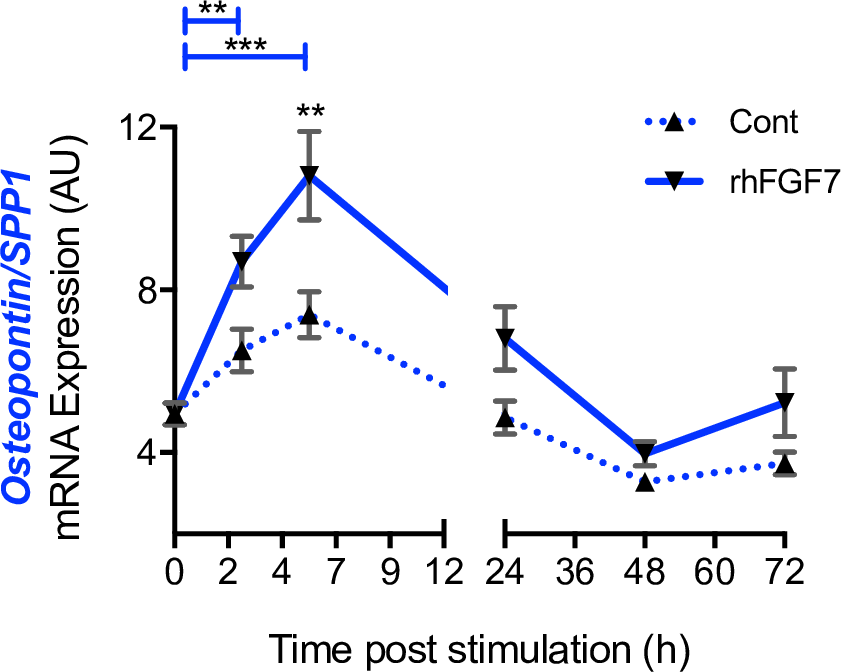

Supplement: S9 Fig — RT-qPCR time course of rhFGF7 response in HepaRG cells (day 13). Changes in osteopontin/SPP1 are compared to vehicle-treated cont. Data information: underlying data are available in S2 Data. Data are presented as mean + SEM. Data are presented as mean + SEM. *P < 0.05, **P < 0.01, and ***P < 0.001. Two-way ANOVA was used to compare means. Significance P values were calculated using Tukey's multiple comparison test. cont, control; Fgf7, fibroblast growth factor 7; rhFGF7, recombinant human FGF7; RT-qPCR, reverse transcriptase-quantitative PCR; Spp1, secreted phosphoprotein 1. (TIF) [file pbio.2006972.s009.tif]

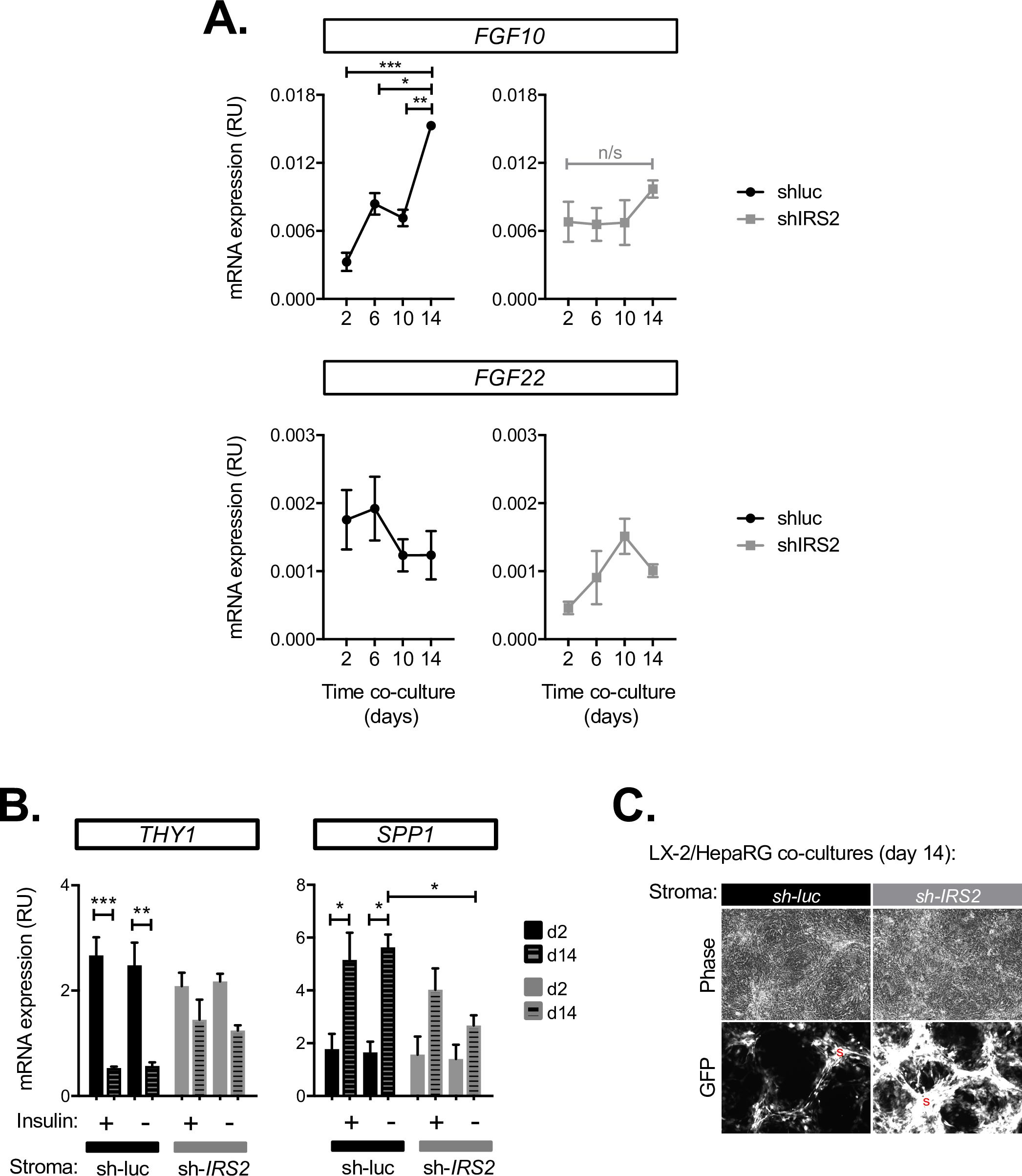

Supplement: S10 Fig — (A) RT-qPCR time course showing changes in FGFR2-IIIb ligand gene expression in LX-2/HepaRG cocultures. Silencing of IRS2 in LX-2 cells resulted in impaired FGF10 induction (above) but had no impact upon FGF22 expression (below) (n = 3). (B) IRS2 was required for time-dependent switching between THY1 and SPP1. Day 2 and day 14 LX-2/HepaRG cocultures maintained in media with (+) or without (−) supplemented insulin were analyzed for mesenchymal genes associated with myofibroblasts (THY1) or LPCs (SPP1) by RT-qPCR. Switching from THY1 to SPP1 was observed in cocultures using control LX-2 stroma (sh-luc) but not in those in which IRS2 was silenced (sh-IRS2) (n = 3). (C) Silencing of IRS2 favored expansion of LX-2 cells in HepaRG coculture. Phase-contrast (Phase) and immunofluorescence images (GFP) taken on day 14 of LX-2/HepaRG coculture. Stromal expansion within the cocultures (s) was tracked by lentiviral coexpression of GFP in control (sh-luc) or IRS2-deficient (sh-IRS2) LX-2 cells. (Images are representative of n = 3.) Data information: underlying data are available in S2 Data. Data are presented as mean + SEM. *P < 0.05, **P < 0.01, and ***P < 0.001. Two-way ANOVA was used to compare means. Significance P values were calculated using (A) Tukey's or (B) Sidak’s multiple comparison tests. Fgfr2-IIIB, Fgf7 receptor; Fgf7, fibroblast growth factor 7; Irs2, insulin receptor substrate 2; RT-qPCR, reverse transcriptase-quantitative PCR; shIRS2, shRNA-targeting IRS2; sh-luc, control luciferase; shRNA, short hairpin RNA; Spp1, secreted phosphoprotein 1; Thy1, Thy-1 cell surface antigen. (TIF) [file pbio.2006972.s010.tif]
