# Supplementary material for: Insulin resistance disrupts epithelial repair and niche-progenitor Fgf signaling during chronic liver injury
Source: PLoS Biol. 2019 Jan 29;17(1):e2006972. doi: 10.1371/journal.pbio.2006972 (PMC6368328; doi:10.1371/journal.pbio.2006972)
Supplement: S1 Table — (DOCX) [file pbio.2006972.s011.docx]

| **Name** | **Lentiviral Vector** | **Source** |
| --- | --- | --- |
| GFP control | rLV-EF1-GFP | Vectalys |
| EF1-mIrs2 | rLV-EF1-mIrs2 | Vectalys |
| pIRS2-GFP | rLV-pIRS2-GFP | Vectalys |
| pAPOA2-GFP | rLV-pAPOA2-GFP | Vectalys |
| shRNA-luc | SMARTvector 2.0 Luciferase control | Dharmacon |
| shRNA-scrambled | SMARTvector 2.0 non-targeting control | Dharmacon |
| shRNA-IRS2 | SMARTvector 2.0 SH-003554-03-10 | Dharmacon |
