# Supplementary material for: Insulin resistance disrupts epithelial repair and niche-progenitor Fgf signaling during chronic liver injury
Source: PLoS Biol. 2019 Jan 29;17(1):e2006972. doi: 10.1371/journal.pbio.2006972 (PMC6368328; doi:10.1371/journal.pbio.2006972)
Supplement: S2 Table — ICC, immunocytochemistry; IHC, immunohistochemistry. (DOCX) [file pbio.2006972.s012.docx]

| **Antibody name** | **Host** | **Company** | **Dilution** | | |
| --- | --- | --- | --- | --- | --- |
|  |  |  | **Western** | **ICC** | **IF** |
| Anti-Albumin | Mouse | Sigma Aldrich (A6684) |  | 1:1000 |  |
| Anti-β-actin | Mouse | Sigma Aldrich (A1978) | 1:40000 |  |  |
| Anti-CD3 | Rabbit | Agilent Dako (IR503) |  |  | 1:1 |
| Anti-CD45 | Rat | R&D Systems (MAB114-SP) |  |  | 1:200 |
| Anti-cCaspase3 (Asp175) | Rabbit | Cell Signalling Technology (cs9664) | 1:500 |  | 1:800 |
| Anti-CYP34A | Rabbit | Millipore (AB1254) |  | 1:500 |  |
| Anti-Elastin | Mouse | Santa Cruz Biotechnology (sc-58756) |  |  | 1:100 |
| Anti-Epcam | Rabbit | Abcam (ab32392) |  |  | 1:200 |
| Anti-Erk1/2 | Rabbit | Cell Signaling (9271) | 1:1000 |  |  |
| Anti-FGF7 | Mouse | R&D systems (MAB251-100) |  |  | 1:200 |
| Anti-FGFR2 | Rabbit | Sigma Aldrich (F0300) |  | 1:500 | 1:300 |
| Anti-Gfap | Rabbit | DAKO (Z0334) | 1:1000 |  | 1:250 |
| Anti-human HNF4α | Rabbit | Santa Cruz Biotechnology clone H-171 (sc-8987) |  | 1:50 |  |
| Anti-IRS2 | Mouse | Santa Cruz Biotechnology  B-5 (sc-390761) | 1:500 |  |  |
| Anti-Ki67 | Rabbit | Abcam (ab15580) |  |  | 1:800 |
| Anti-Krt19 | Mouse | Santa Cruz Biotechnology clone BA17 (sc-53258) |  |  | 1:300 |
| Anti-Mic1-1c3 | Rat | Thermofisher Pierce antibodies MA5-16136 |  |  | 1:100 |
| Anti-mouse HNF4α | Goat | Santa Cruz Biotechnology clone C-19 (sc-6556) |  |  | 1:200 |
| Anti-p53 | Mouse | Santa Cruz (sc71819) | 1:500 |  |  |
| Phalloidin  (Alex. Fluor 647) |  | Invitrogen (A22287) | 1:50 |  |  |
| Anti-phospho-Erk1/2 (Tyr 204) | Mouse | Santa Cruz Biotechnology clone E-4 (sc-7383) | 1:500 |  |  |
| Anti-Sox9 | Rabbit | Millipore (AB5535) |  |  | 1:100 |
| Anti-Spp1 | Goat | R&D systems AF808 |  |  | 1:200 |
| Anti-Thy1 | Mouse | Santa Cruz Biotechnology (sc-53456) |  |  | 1:100 |
| Anti-Vimentin | Mouse | Santa Cruz Biotechnology clone E-5(sc-373717) |  | 1:300 | 1:200 |
| Anti-α sma | Mouse | Agilent Dako clone 1A4 (IR611) |  | 1:1 | 1:1 |
| Anti-α sma | Rabbit | Abcam (ab5694) |  |  | 1:100 |
| Anti-β Catenin | Mouse | Agilent Dako (IR702) |  | 1:1 |  |
