# Supplementary material for: Insulin resistance disrupts epithelial repair and niche-progenitor Fgf signaling during chronic liver injury
Source: PLoS Biol. 2019 Jan 29;17(1):e2006972. doi: 10.1371/journal.pbio.2006972 (PMC6368328; doi:10.1371/journal.pbio.2006972)
Supplement: S3 Table — RT-qPCR, reverse transcriptase-quantitative PCR. (DOCX) [file pbio.2006972.s013.docx]

**Supplementary Table 3 – List of primers used for RT-qPCR**

| **Target** |  | **Forward Primer** | **Reverse Primer** |
| --- | --- | --- | --- |
| ACTA2 | H | 5’-CAGTGGCCATCTCATTTTCA-3’ | 5’-CTGAGCGTGGCTATTCCTTC-3’ |
|  | m | 5’-GACACCACCCACCCAGAGT-3’ | 5’-ACATAGCTGGAGCAGCGTCT-3’ |
| ALB | H | 5'-TGTTGATTGCCTTTGCTCAG-3' | 5'-GCAGTCAGCCATTTCACCAT-3' |
| APOA2 | H | 5'-TCGCAGCAACTGTGCTACTC-3' | 5'-TCTGGGCTCTTGACCTTCTC-3' |
| COL1A1 | H | 5’-TGGTGACAAGGGTGAGACAG-3’ | 5’-CTCCAGAGGGACCTTGTTCA-3’ |
| COL3A1 | H | 5’-GGTGAGCCTGGTAAGAATGG-3’ | 5’-CTTGCCATCTTCGCCTTTAG-3’ |
| CTGF | m | 5’- GCAGACTGGAGAAGCAGAGC-3’ | 5’-GCTTGGCGATTTTAGGTGTC-3’ |
| ELN | m | 5’-TTGCTGATCCTCTTGCTCA-3’ | 5’-GCCCCTGGATAATAGACTC-3’ |
| EPCAM | m | 5’-AACACAAGACGACGTGGACA-3’ | 5’-CCGTTCACTCTCAGGTCCAT-3’ |
| ESRP1 | m | 5’-GAGGCACAAACATCACATGG-3’ | 5’-AACTGGGCTACCTCATTGGA-3’ |
| ESRP2 | H | 5’-TGGAGACAGATGCCACAGAG-3’ | 5’-GGGCTTCGAAAACAATTGAC-3’ |
|  | m | 5’-GAGGCACAAACATCACATGG-3’ | 5’-AACTGGGCTACCTCATTGGA-3’ |
| FGF7 | H | 5’-TGCAATGAACAAGGAAGGAA-3’ | 5’-CCGTTGTGTGTCCATTTAGC-3’ |
|  | m | 5’-TTGACAAACGAGGCAAAGTG-3’ | 5’-CCCTTTGATTGCCACAATTC-3’ |
| FGF10 | H | 5’-CCATGAACAAGAAGGGGAAA-3’ | 5’-TGCTGCCAGTTAAATGATGC-3’ |
|  | m | 5’-GAGAAGAACGGCAAGGTCAG-3’ | 5’-TTGCTGTTGATGGCTTTGAC-3’ |
| FGF22 | H | 5’-CACGGCCAGGACAGCATC-3’ | 5’-TAGAAGCCTGAGGACACTGC-3’ |
|  | m | 5’-GACACGACGGCACCAACT-3’ | 5’-AGGCCCTTCAAGACGAGAC-3’ |
| FGFR2 | H | 5’-AAGCTGCTGAAGGAAGGACA-3’ | 5’-TGCTTGAACGTTGGTCTCTG-3’ |
| FGFR2-IIIb | H | 5’-TGCTGGCTCTGTTCAATGTG-3’ | 5’-GGCGATTAAGAAGACCCCTA-3’ |
|  | m | 5’-AAGGTTTACAGCGATGCCCA-3’ | 5’-AGAGCCAGCACTTCTGCATT-3’ |
| FGFR2-IIIc | H | 5’-ACACCACGGACAAAGAGATT-3’ | 5’-GGCGATTAAGAAGACCCCTA-3’ |
| GAPDH | m | 5’-CGTCCCGTAGACAAAATGGT-3’ | 5’-TCGTTGATGGCAACAATCTC-3’ |
| GFAP | m | 5’-TGCAAGAGACAGAGGAGTGG-3’ | 5’-GGCGATAGTCGTTAGCTTCG-3’ |
| IRS2 | H | 5’-GTTTCCAGAAGCAGCCAGAG-3’ | 5’-TGAAATGGATGCATCGTACC-3’ |
|  | m | 5’-CAGCTGCCCAGTAGCGTTGCG-3’ | 5’-CCCGCTTGGCTCTCTAGGCG-3’ |
| KIT | m | 5’-TGCCAACCAAGACAGACAAG-3’ | 5’-AGCAAATCATCCAGGTCCAG-3’ |
| MMP9 | m | 5’-CATTCGCGTGGATAAGGAGT-3’ | 5’-TCACACGCCAGAAGAATTTG-3’ |
| MYC | m | 5’-AGTGCTGCATGAGGAGACAC-3’ | 5’-GGTTTGCCTCTTCTCCACAG-3’ |
| PTPRC | m | 5’-ATCAAAGATGCCCGAAAGCC-3’ | 5’- TGGACCCTGCATCTCCATTT-3’ |
| RPL19 | H | 5'-CGAATGCCAGAGAAGGTCAC-3' | 5'-CCATGAGAATCCGCTTGTTT-3' |
| SPP1 | H | 5’-GCCGAGGTGATAGTGTGGTT-3’ | 5’-CCATGTGTGAGGTGATGTCC-3’ |
|  | m | 5’-GCTTGGCTTATGGACTGAGG-3’ | 5’-GACTCACCGCTCTTCATGTG-3’ |
| TGFβ | H | 5’-TTTTGATGTCACCGGAGTTG-3’ | 5’-TGCAGTGTGTTATCCCTGCT-3’ |
|  | m | 5’-TGGAGCAACATGTGGAACTC-3’ | 5’-CGTCAAAAGACAGCCACTCA-3’ |
| THY1 | H | 5’-AGCATCGCTCTCCTGCTAA-3’ | 5’-GTATTCTCATGGCGGCAGTC-3’ |
|  | m | 5’-GGGCGACTACTTTTGTGAGC-3’ | 5’-TCTGAACCAGCAGGCTTATG-3’ |
| TIMP1 | m | 5’-CATGGAAAGCCTCTGTGGAT-3’ | 5’-AAGAAGCTGCAGGCACTGAT-3’ |
| VIM | m | 5’-GTACCGGAGACAGGTGCAGT-3’ | 5’-CAGCTTCAAGGGCAAAATTC-3’ |
